# Flexoelectric Elastomer Enabled by Miscibility‐Driven Succinonitrile Molecular Rotation

**DOI:** 10.1002/advs.202520317

**Published:** 2025-11-07

**Authors:** Moonseok Jang, Bitgaram Kim, Ji‐Hun Seo

**Affiliations:** ^1^ Department of Materials Science and Engineering Korea University 145 Anam‐ro, Seongbuk‐gu Seoul 02841 Republic of Korea; ^2^ Pritzker School of Molecular Engineering University of Chicago Chicago IL 60637 USA

**Keywords:** elastomer, flexoelectricity, miscibility, polymer moieties, succinonitrile

## Abstract

Plastic crystals exhibit long‐range positional order alongside orientational disorder, positioning them between crystalline solids and liquids. Although their centrosymmetric structure suppresses intrinsic piezoelectric and ferroelectric effects, they can exhibit flexoelectric behavior under strain gradient. Succinonitrile (SN), a highly polar plastic crystal, is a promising candidate for such applications due to its dynamic molecular nature. While previous studies focused on the rotational dynamics of pure and salt‐doped SN, this study explores how covalently crosslinked polymer networks affect the rotational dynamics and resulting flexoelectric properties of SN. Systematic variation of polymer–SN miscibility demonstrates that the microstructure and SN molecular mobility critically influence electromechanical coupling. Among the tested systems, the SN_MMA composite achieves the highest performance, generating 2.79 V and 0.082 µA cm^–^^2^ under finger tapping, consistent with its highest flexoelectric coefficient of 22.8 nC m^−1^ measured under three‐point bending. Morphological observation and dielectric analyses, supported by DFT calculations, reveal that the highest electromechanical coupling performance of SN_MMA arises from the combined effects of interfacial stress localization and preserved orientational freedom. These findings establish design principles for soft, non‐piezoelectric materials capable of efficient mechanical‐to‐electrical energy conversion.

## Graphical abstract

A flexoelectric material platform is developed by embedding polar plastic crystals into polymer networks. By tuning the microstructure and preserving molecular rotation, strain gradient‐induced polarization is achieved. The resulting elastomer shows scalable and tunable electromechanical output under mechanical deformation, offering a new design route for non‐piezoelectric energy harvesting and soft sensing applications.

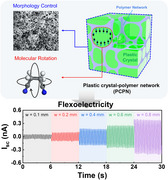

## 1 Introduction

Plastic crystals are a unique class of mesophase materials that exhibit long‐range positional order while having dynamic orientational disorder.^[^ ^1^, ^2^, ^3^ ^]^ Upon heating above the plastic crystal transition temperature (*T_pc_*), plastic crystals lose their orientational order as they transform from an ordered crystalline phase to a plastic crystalline phase. Due to the randomness of the molecular orientation, each molecule occupies an isotropic spatial distribution, effectively occupying a nearly spherical volume, impeding close packing and increasing intermolecular distances compared to typical crystalline solids.^[^ ^4^ ^]^ The loose molecular packing enables facile slippage of planes and migration of dislocations, resulting in soft, ductile, flexible, and processable characteristics.^[^ ^5^, ^6^ ^]^ Owing to their unique combination of mechanical softness and structural order, plastic crystals have recently emerged as promising materials for diverse applications such as flexible electronics,^[^ ^7^, ^8^ ^]^ solid‐state electrolytes,^[^ ^9^, ^10^, ^11^ ^]^ and barocalorics.^[^ ^12^, ^13^ ^]^

Recently, the potential of plastic crystals as electromechanically responsive materials has been pointed out, given their ability to address the limitations of conventional ordered crystalline materials, such as insufficient mechanical flexibility and limited processability.^[^ ^3^ ^]^ Following the first discovery of a ferroelectric, organic ionic plastic crystal (OIPC) by Harada's group^,[^ ^14^ ^]^ a broad range of ferroelectric and piezoelectric plastic crystals have been explored over the past decade.^[^ ^4^, ^7^, ^15^, ^16^ ^]^ However, at temperatures above *T_pc_*, increased molecular mobility and effective spherical molecular geometry lead to the formation of centrosymmetric cubic crystal structures, which inhibit both ferroelectricity and piezoelectricity. Consequently, the electromechanical responses of plastic crystals have been strictly confined to the low‐temperature, brittle, ordered phase, preventing full utilization of their mechanical advantages and hindering practical applications in flexible electronics. To the best of our knowledge, the electromechanical response of plastic crystals above the *T_pc_* has not yet been investigated.

**Figure 1:**
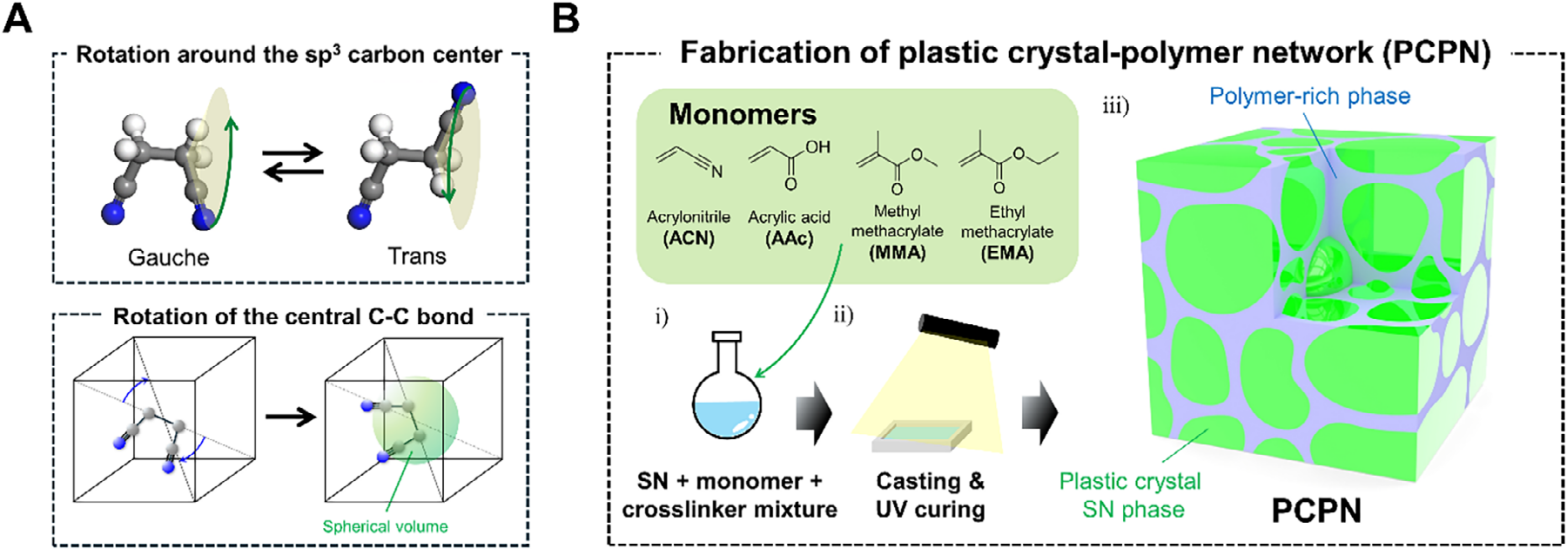
a) Schematic illustration of the rotational degrees of freedom in SN. b) Schematic of the fabrication process for PCPN samples.

Flexoelectricity, the electromechanical coupling effect between electric polarization and mechanical strain gradient, offers a promising pathway to derive electromechanical responses in such materials. Unlike piezoelectricity, the flexoelectric effect can occur in all crystallographic point groups, as inhomogeneous deformation breaks local symmetry and induces charge displacement.^[^ ^17^, ^18^ ^]^ In this context, we investigate the flexoelectric electromechanical coupling behavior of succinonitrile (SN), a representative plastic crystal with a chemical structure of N≡C–CH_2_–CH_2_–C≡N, in its centrosymmetric phase above the plastic crystal transition temperature. Extensive studies on the molecular rotation dynamics and structure of SN have revealed two distinct types of rotational motion of SN in its plastic crystalline phase (**Figure** **1**a).^[^ ^19^, ^20^, ^21^ ^]^ The first is the rotation of the polar nitrile groups around the central sp^3^‐hybridized carbon atom, which enables dynamic conformational equilibrium between two gauche and one trans conformation. The second is the molecular reorientation around the central C─C bond, which aligns with one of the four‐fold diagonal axes of the lattice. Despite its linear structure and strong polarity, SN exhibits a broad plastic crystal phase over a low temperature range (−35 to 62 °C) due to its orientational freedom.^[^ ^20^ ^]^ Such rotational mobility is further expected to enhance the flexoelectric performance of SN by introducing additional orientational polarization through the partial alignment of highly polar molecular dipoles.

However, the intrinsic plasticity of SN limits its application as an electromechanical transducer, as it lacks the elasticity required for reversible deformation. In addition, inspired by previous studies, we considered that introducing heterogeneous microstructures, rather than a homogeneous SN phase, could further enhance flexoelectricity by increasing dielectric interface areas and amplifying surface‐induced polarization effects.^[^ ^22^, ^23^ ^]^ Therefore, we systematically designed a series of plastic crystal–polymer network (PCPN) hybrids by incorporating SN into covalently crosslinked polymer matrices via one‐pot free radical polymerization of SN, monomer, and crosslinker mixtures (Figure 1b). Employing polymer networks with varying miscibility with SN led to distinct microstructures, and the resulting mechanical properties were evaluated. Furthermore, the interaction energies between SN and the respective polymers were assessed using dispersion‐corrected density functional theory (DFT‐D) calculations. The rotational dynamics of the SN phase, strongly influenced by interactions with the polymer network, were characterized by broadband dielectric spectroscopy and ^13^C spin‐lattice (T_1_) relaxation analysis. Finally, the flexoelectric performance of the PCPNs was evaluated, and the effects of microstructure and rotational dynamics on their electromechanical response were analyzed. This study introduces a new strategy for achieving flexoelectric electromechanical coupling in plastic crystalline systems through the synergistic integration of dynamic molecular components such as SN within structurally tunable polymer networks.

## 2 Results and Discussion

### 2.1 Characterization of PCPN Elastomers

Four monomers with different polarity and functional groups—acrylic acid (AAc), acrylonitrile (ACN), ethyl methacrylate (EMA), and methyl methacrylate (MMA)— were selected to form the polymer networks of the PCPN system (Figure 1b). Due to its weak intermolecular interactions and polar nitrile groups, SN exhibited excellent solubility in the acrylate monomers (Figure S1 and Table S1, Supporting Information). This compatibility enables the formation of a uniform mixture with high SN content, facilitating efficient one‐pot bulk free radical polymerization without additional solvents (Figure 1b). The PCPN films were prepared with a fixed SN content of 58.3 wt.%, corresponding to the maximum solubility of SN in EMA. The fabricated films were labeled as SN_x, where x represents the used monomer. Successful polymerization of monomers in the presence of SN was confirmed by FT‐IR spectroscopy (Figure S2, Supporting Information), where the characteristic C≡N stretching band of SN at 2253 cm^−1^ remained visible, while the IR bands at 1637 cm^−1^ (C═C stretch), associated with the double bonds of the acrylic monomer, disappeared after polymerization.^[^ ^24^ ^]^

**Figure 2:**
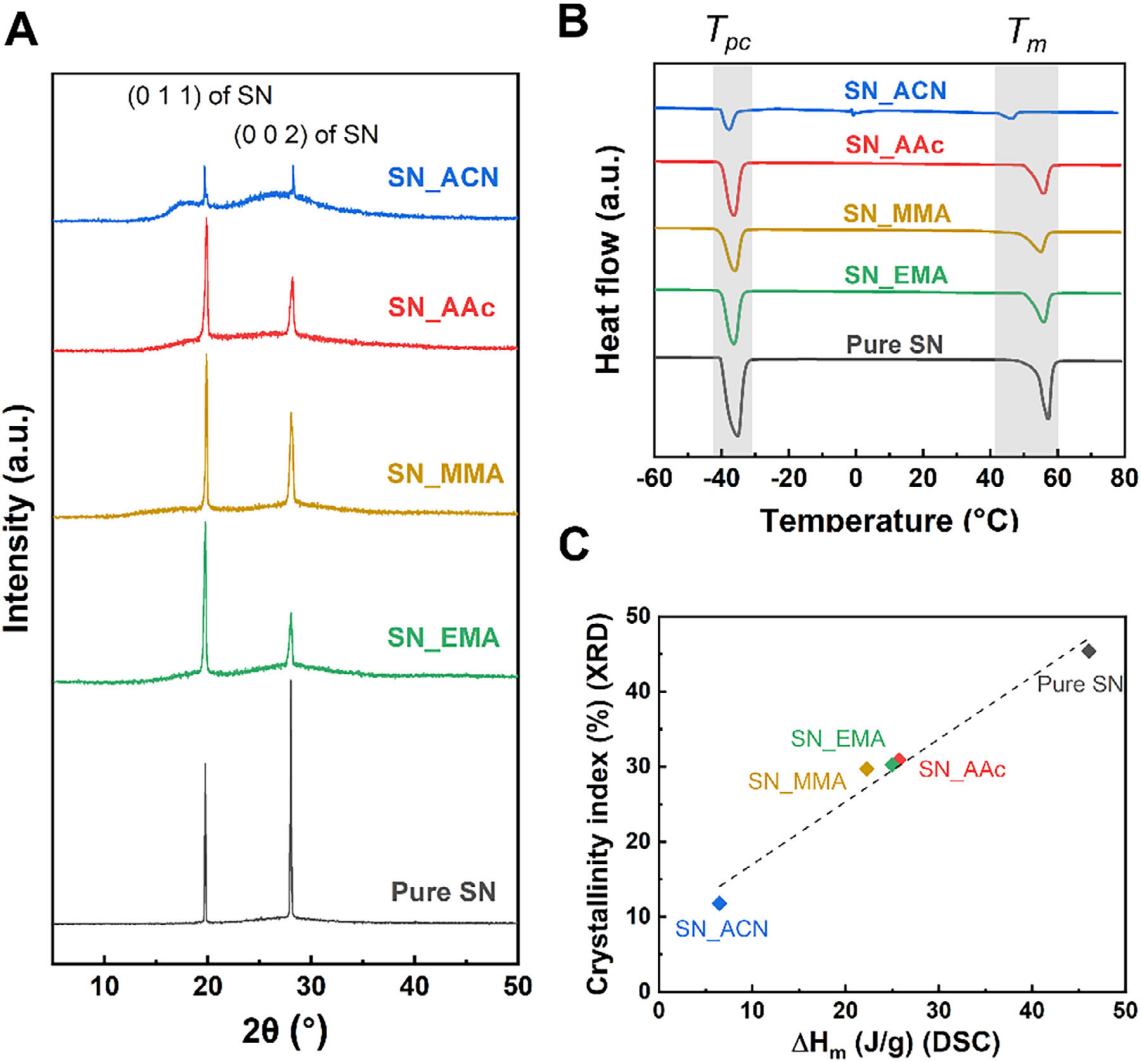
a) XRD spectra and b) DSC traces (exo up) of PCPNs and pure SN. c) Crystallinity and *ΔH_m_* derived from XRD and DSC analyses, respectively.

As polymerization proceeded, the formation of a crosslinked network reduced the solubility of SN within the matrix, triggering polymerization‐induced phase separation (PIPS).^[^ ^9^, ^25^ ^]^ The PIPS behavior resulted in the segregation of SN into two distinct domains: a polymer phase plasticized by amorphous SN and a separate crystalline SN phase. **Figure** **2**a shows the powder XRD (PXRD) patterns of the PCPN samples and pure SN. All PCPNs exhibited the characteristic (011) and (002) peaks of plastic crystalline SN, confirming the partial retention of its ordered phase within the networks.^[^ ^11^, ^26^ ^]^ Among the samples, SN_ACN displayed an additional broad and shifted peak, indicating significant disruption of SN crystallinity by the ACN matrix. In contrast, SN_AAc, SN_EMA, and SN_MMA showed sharper diffraction peaks closely matching those of pure SN, suggesting better preservation of the crystalline phase. These crystallinity differences are further supported by DSC thermograms in Figure 2b. All PCPNs exhibited two characteristic endothermic peaks of SN, corresponding to the solid–solid phase transition from the crystalline to plastic crystal phase (*T_pc_*), and the melting transition (*T_m_*).^[^ ^11^, ^27^ ^]^ To compare the crystalline SN content among the PCPNs, the enthalpy of fusion (*ΔH_m_*) values were analyzed and correlated with the XRD‐derived crystallinity index, as presented in Figure 2c. SN_ACN had the lowest *ΔH_m_*, supporting the interpretation that the ACN network strongly suppressed SN crystallization. In contrast, networks based on AAc, EMA, and MMA exhibited higher enthalpy values, consistent with their sharper PXRD peaks and higher crystalline content. A clear linear correlation between *ΔH_m_* and the crystallinity index was observed across all PCPNs, indicating the consistency and reliability of the thermal and structural analyses. Collectively, these results demonstrate that the chemical structure of the polymer network significantly affected the phase behavior of embedded SN. The ACN matrix caused the most severe disruption of crystallinity, whereas AAc, EMA, and MMA allowed more effective retention of the plastic crystalline phase.

### 2.2 Effect of Polymer‐SN Miscibility on Morphological Properties of PCPNs

**Figure 3:**
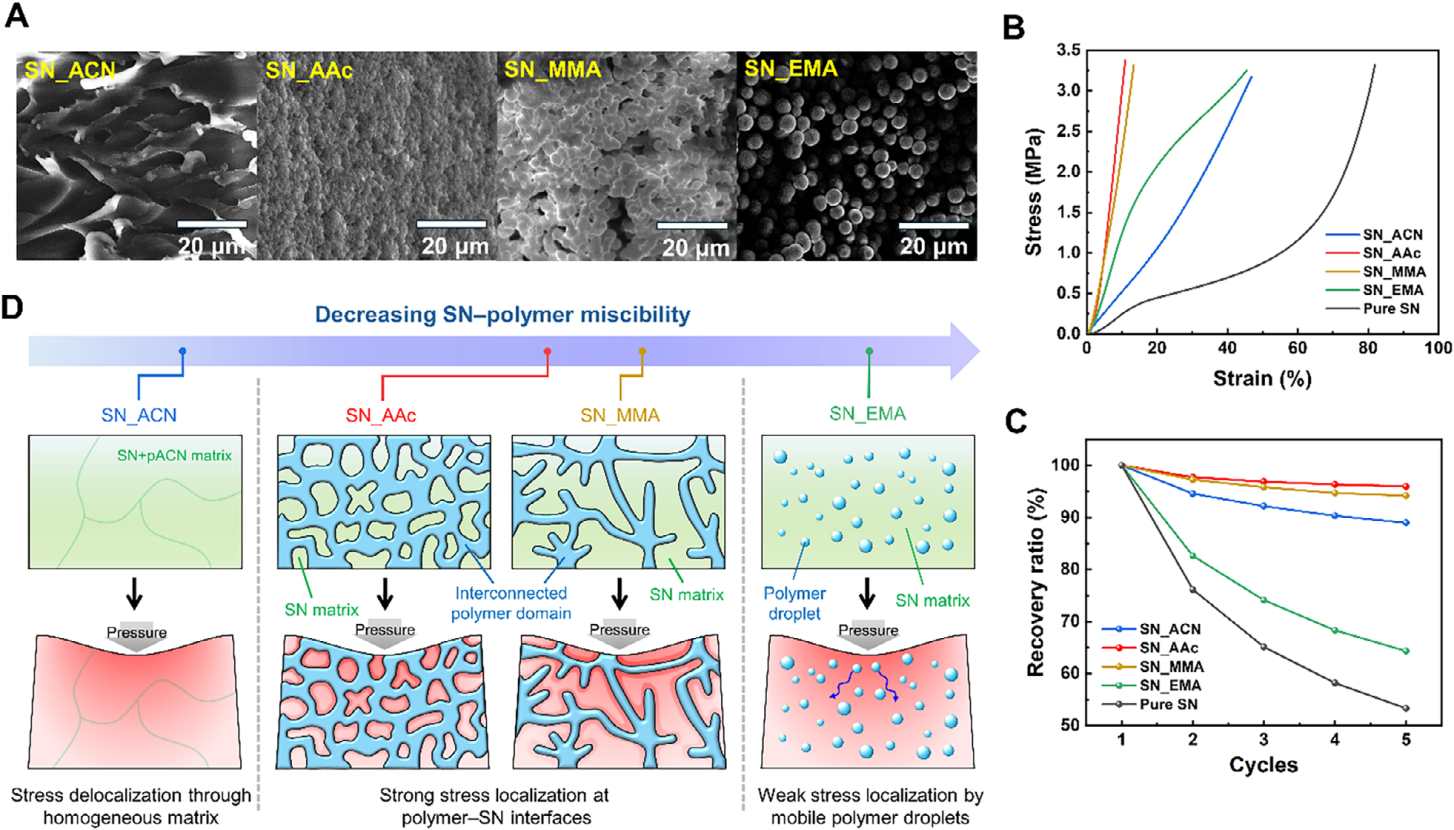
a) Cross‐sectional SEM images of PCPN samples. b) Stress‐strain curves from uniaxial compression tests of PCPNs and c) recovery ratio of tensile strength in PCPNs under repeated 10% strain cycles. d) Schematic illustration of PCPN morphology and stress distribution under applied pressure by increasing SN‐polymer miscibility.

To gain further insight into the phase separation behavior of PCPNs, cross‐sectional SEM micrographs were analyzed (**Figure** **3**a). SN_ACN displayed a homogeneous network with no visible phase boundaries, consistent with its low crystallinity as revealed by PXRD and DSC (Figure 2a,b), and the slightly transparent appearance observed in optical images (Figure S3, Supporting Information). In contrast, the other three PCPNs exhibited inhomogeneous microstructures, each with distinct morphologies. These differences are primarily attributed to the variation in miscibility between SN and the respective monomers, which influence the pathway and dynamics of phase separation during polymerization.^[^ ^28^, ^29^ ^]^ Specifically, SN_AAc and SN_MMA both formed interconnected polymer networks, but with different structural features reflective of their relative compatibility with SN. SN_AAc, which exhibited relatively better miscibility, formed a fine and compact network, whereas SN_MMA, with lower miscibility, developed a coarse, dendritic morphology containing prominent non‐polymer regions. Meanwhile, SN_EMA showed poor miscibility, resulting in a well‐defined phase separation, with isolated, droplet‐like polymer domains (3–10 µm) embedded within the SN‐rich matrix. The formation of these diverse microstructures can also be understood by considering the relative rates of polymerization and phase separation. As polymerization proceeded, the molecular weight of the growing polymer chains increased, gradually reducing their miscibility with SN. Once the miscibility threshold was exceeded, phase separation initiated. When phase separation progressed faster than polymerization, polymer‐rich regions became confined into isolated droplets, where polymerization continued independently within each domain.^[^ ^29^, ^30^ ^]^ Conversely, when polymerization outpaced phase separation, the growing polymer chains remained interconnected, leading to the formation of a continuous network. To confirm the differences in microstructure, the PCPNs were subjected to high pressure (>1 GPa) to induce substantial deformation, and the cross‐sectional SEM images of the deformed PCPNs were compared (Figure S4, Supporting Information). Despite the high compressive force, the shape and size of the polymer droplets in SN_EMA remained unchanged. Rather than deforming, the droplets shifted along the direction of the applied pressure and became more densely packed compared to the uncompressed sample. In contrast, SN_AAc and SN_MMA exhibited pronounced deformation of their polymer networks, further confirming that these systems possess continuous and mechanically integrated structures (Figure 3a and Figure S4, Supporting Information).

To investigate the effect of structural differences on mechanical behavior, uniaxial compression tests were performed on cylindrical disk samples of PCPNs and pure SN (Figure 3b). Among the samples, SN_AAc exhibited the highest compressive modulus of 16.9 MPa, followed by SN_MMA at 14.0 MPa. Both samples exhibited interconnected polymer networks in the SEM analysis. In comparison, pure SN had a modulus of 0.945 MPa, where the ≈18‐fold increase in modulus for SN_MMA indicates that the heterogeneous polymer network enhanced mechanical robustness, acting as a scaffold that effectively resisted compressive deformation.^[^ ^30^, ^31^ ^]^ In contrast, SN_EMA exhibited a modulus of 8.56 MPa, indicating that its dispersed polymer microdroplets provided limited reinforcement compared to the continuous network structures. SN_ACN showed the lowest modulus among the PCPNs at 5.50 MPa, likely due to the lack of structural heterogeneity that contributes to mechanical reinforcement, instead exhibiting heavily plasticized, solvated polymer chains, as revealed by the XRD analysis.^[^ ^30^, ^31^, ^32^ ^]^ To further support the mechanical behavior of PCPNs, the recovery ratios of tensile strength after five cycles of 10% strain are presented in Figure 3c. SN_AAc and SN_MMA exhibited high recoverability of 96.0% and 94.2%, respectively, consistent with the earlier discussion that their interconnected scaffold‐like structures can effectively withstand compressive loading. In contrast, SN_EMA displayed poor recoverability of 64.3%, similar to that of pure SN (53.3%), supporting the interpretation that the polymer phase of SN_EMA is dispersed as isolated droplets within the SN matrix, resulting in highly plastic deformation behavior. SN_ACN exhibited moderate recoverability of 89.0%, which can be attributed to the lack of a plastic‐deforming SN phase and the presence of a homogeneous, plasticized polymer network phase. These results suggest that microstructural morphology plays a crucial role in determining the mechanical properties of PCPN elastomers.

To provide further insight into the influence of microstructure on electromechanical properties, representative morphologies of the PCPNs are presented in Figure 3d, ordered by decreasing miscibility between SN and the polymer network. Inducing substantial strain gradients at the plastic crystal interface is essential for generating polarization in SN, contributing to enhanced flexoelectric or surface piezoelectric effects.^[^ ^33^ ^]^ Interconnected, heterogeneous polymer networks, such as those observed in SN_AAc and SN_MMA, are expected to support more efficient stress transfer by forming continuous mechanical pathways. These structures promoted localized strain concentration at the SN interface, favorable for enhancing polarization.^[^ ^34^ ^]^ In contrast, fully phase‐separated droplet‐like morphologies, as seen in SN_EMA, provided limited mechanical reinforcement. The dispersed polymer domains tended to migrate under applied stress, leading to stress relaxation and plastic deformation. Similarly, SN_ACN lacked the structural heterogeneity required for stress localization, potentially diminishing the strain‐induced polarization. From this perspective, we hypothesize that SN_AAc and SN_MMA are morphologically advantageous for flexoelectric performance, while SN_EMA and SN_ACN are expected to be less effective.

### 2.3 Effect of Polymer‐SN Miscibility on Rotational Freedom of SN

In ordered crystalline materials, where molecular dipole orientations are fixed, the polarization induced by a strain gradient in their centrosymmetric lattices arises solely from perturbations of positional order and the breaking of local symmetry.^[^ ^17^, ^35^ ^]^ In contrast, plastic crystals are expected to exhibit an additional contribution from orientational polarization, as strain‐induced preferential alignment of dipoles could further enhance the overall polarization within the plastic crystal matrix. The orientational relaxation dynamics of plastic crystals are primarily governed by α‐relaxation, which corresponds to molecular reorientation. This relaxation spans an exceptionally broad and temperature‐dependent timescale, ranging from 10^2^ to 10^−10^ s.^[^ ^2^ ^]^ However, SN exhibits a distinct orientational behavior compared to other common plastic crystals. Its experimentally measured density exceeds the value calculated for assuming freely rotating molecules, suggesting shorter intermolecular distances and preferred molecular orientations. Thus, the rotation of a single SN molecule requires cooperative motion of adjacent molecules, leading to a higher activation energy.^[^ ^36^ ^]^ Moreover, SN exhibits an additional rotational mode, in which the rotation of nitrile groups induces dynamic conformational switching between trans and gauche states. This motion occurs on a faster timescale (10^−10^ to 10^−12^ s), as rotation of the nitrile group is sterically less hindered than that of the entire molecule.^[^ ^36^, ^37^ ^]^ Therefore, a comprehensive analysis encompassing both short‐ and long‐timescale relaxation regimes, together with polymer–SN interaction energies, is necessary to fully elucidate the orientational dynamics of the SN phase within the polymer network.

**Figure 4:**
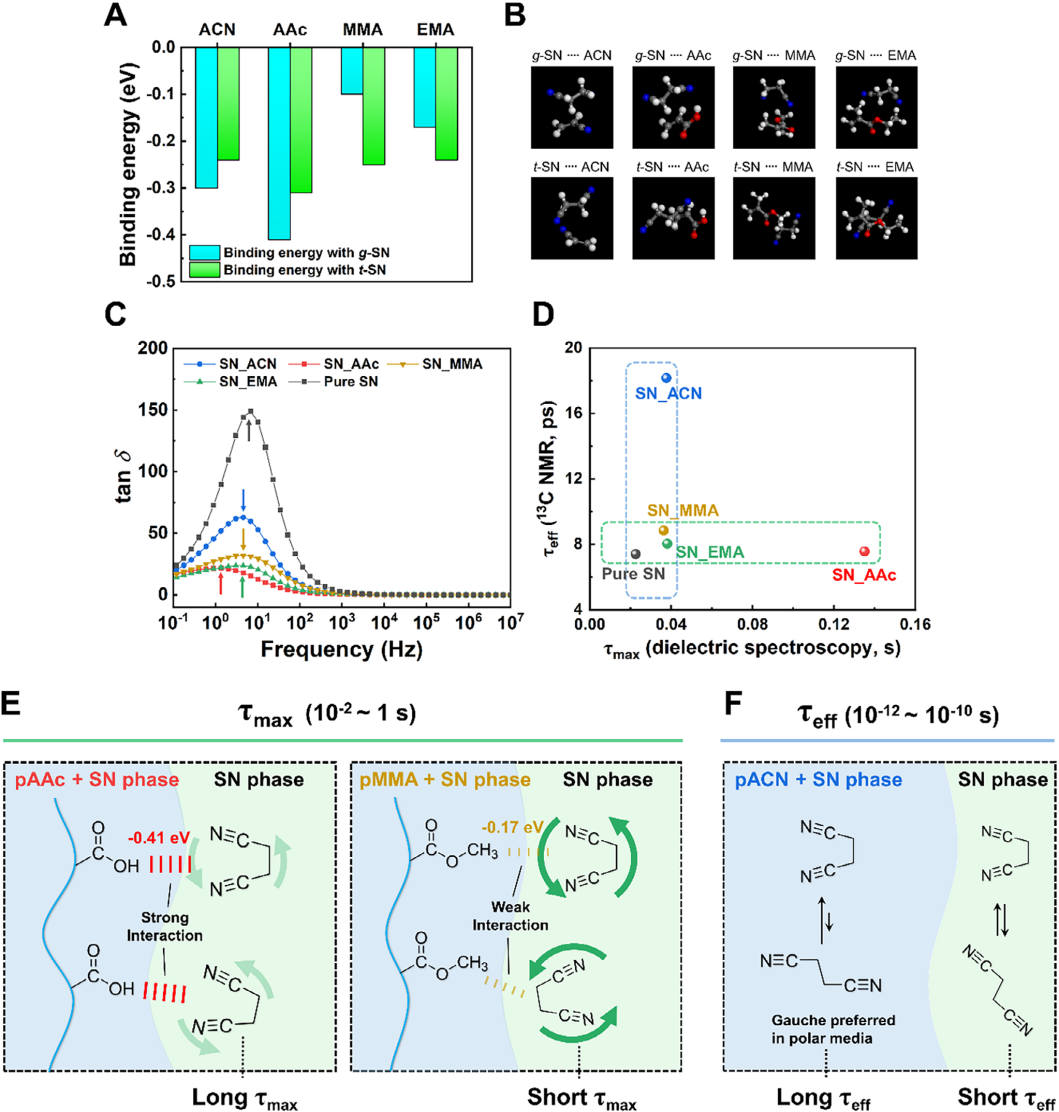
a) Binding energy between SN conformers and monomers calculated via DFT‐D simulations. b) Optimized geometries obtained from DFT‐D calculations. c) tan δ obtained from frequency‐dependent dielectric spectroscopy of PCPN samples, with arrows indicating the relaxation peaks. d) Correlation plot of relaxation times, showing *τ_max_* obtained from dielectric spectroscopy (*x*‐axis) and *τ_eff_* obtained from ^13^C NMR spin–lattice relaxation (*y*‐axis). Schematic illustration of the proposed relaxation mechanisms, e) *τ_max_* and f) *τ_eff_*.

First, DFT‐D calculations were performed to determine the interaction energies between SN and the representative functional groups of each monomer (**Figure** **4**a,b). Both gauche and trans conformers of SN were considered, as they correspond to local energy minima. Geometry optimizations preserved the original conformation in each case, confirming their energetic stability. Accordingly, binding energies were calculated for each monomer interacting with both conformers to evaluate their relative interaction strengths. AAc exhibited the strongest interaction with gauche SN (*g*‐SN), with a binding energy of –0.41 eV attributed to hydrogen bonding between the hydroxyl group in the carboxylic acid and the nitrile group of SN.^[^ ^38^ ^]^ ACN showed the next highest binding energy at –0.30 eV, likely arising from dipole–dipole interactions between the nitrile groups of both molecules. In contrast, the less polar monomers EMA and MMA showed significantly lower binding energies with *g*‐SN, measured at –0.17 and –0.10 eV, respectively. This trend is consistent with electrostatic potential maps in Figure S5 (Supporting Information), indicating that the highly polar AAc and ACN preferentially interact with the gauche conformer, while the less polar EMA and MMA exhibit weaker interactions. Binding energies with the trans conformer (*t*‐SN) were comparable across all monomers, as the symmetric *t*‐SN has no net dipole moment and thus interacts primarily through van der Waals forces.^[^ ^39^ ^]^

To experimentally assess the rotational dynamics at the low frequency regime, frequency‐dependent dielectric spectroscopy was conducted, and the resulting loss tangent (tan *δ*) spectra are shown in Figure 4c. At frequencies over 10^3^ Hz, SN molecules remained effectively immobilized as the rapid oscillation of the electric field did not allow sufficient time for dipole reorientation. As the frequency decreased, dipolar relaxation became possible, giving rise to an apparent tan δ peak ranging from 10^−1^ to 10^2^ Hz, corresponding to the α‐relaxation of the SN molecules.^[^ ^40^ ^]^ Considering the peak frequency is inversely proportional to the relaxation time, relaxation times were extracted following Equation (1):

(1) $$f_{max}=\frac{1}{2\pi \tau_{max}}$$

where *f*_max_ is the frequency at maximum tan *δ* and *τ*_max_ is the corresponding relaxation time.^[^ ^21^ ^]^ The relaxation arising from the conformational equilibrium was measured by ^13^C NMR T_1_ relaxation measurements, and the following inversion‐recovery spectra and the T_1_ relaxation times of the samples are shown in Figures S6–S10 (Supporting Information). By assuming that the measurement temperature (298 K) is much higher than the T_1_ minimum at the Larmor frequency, the relaxation can be considered to occur solely through dipole–dipole coupling between adjacent nuclei.^[^ ^41^ ^]^ Accordingly, the relaxation time (*τ*_eff_) for the rotation around the methylene carbon can be expressed as follows:

(2) $$\frac{1}{T_{1}}=2{\left(\frac{\gamma_{C}\gamma_{H}\hbar }{r_{CH}^{3}}\right)}^{2}\tau_{eff}$$

where *γ*_C_ and *γ*_H_ are the gyromagnetic ratios of carbon and hydrogen, respectively, and *r*_CH_ is the C─H bond distance.^[^ ^37^ ^]^ The obtained relaxation times *τ*_max_ and *τ*_eff_ by dielectric spectroscopy and ^13^C NMR, respectively, are presented as a correlation plot at Figure 4d.

Analysis of *τ*_max_ and *τ*_eff_ together with previous studies^[^ ^36^, ^37^, ^39^, ^41^ ^]^ on SN dynamic leads to three considerations: (1) non‐crystalline regions such as defects, grain boundaries, and polymer–SN interfaces are the main contributors of the α‐relaxation of SN; (2) strong polymer–SN interfacial interactions suppress the α‐relaxation of the adjacent SN molecules (Figure 4e); and (3) relaxation of the rotation of nitrile groups occurs faster in the crystalline phase of SN (Figure 4f). The exceptionally long *τ*_max_ of SN_AAc (0.135 s) indicates that the rotational mobility of SN is significantly hindered in the pAAc‐based network, likely due to strong hydrogen bonding interactions. In contrast, the shorter *τ*_max_ observed in SN_EMA (0.0381 s) and SN_MMA (0.0363 s) suggests that SN retained more rotational freedom in these systems, consistent with their weaker binding energies. In the case of the dielectric tan *δ* of SN_ACN (0.0376 s), the larger peak is attributed to the higher amorphous SN content and the increased molecular distance. Although ACN exhibits strong binding energy with SN, the increased intermolecular distance weakens the SN–SN interaction, thereby offsetting the effect of polymer‐SN interactions. In the case of *τ*_eff_, SN_ACN exhibited the longest relaxation time, which is possibly attributed to its preference for the gauche conformer in the polar environment solvated by the pACN matrix. In contrast, SN_AAc, SN_MMA, and SN_EMA showed *τ*_eff_ values similar to that of pure SN, indicating that a larger portion of SN exists in the crystalline phase. We hypothesize that under practical measurement conditions for electricity generation, such as tapping or bending, the difference in *τ*_max_ primarily governs the electromechanical performance, as the measurement frequencies (10^−1^–10^2^ Hz) correspond well to the relaxation time range of *τ*_max_. On the other hand, *τ*_eff_, which has a much shorter time scale (10^−10^–10^−12^ s), is expected to have a negligible effect on the performance differences observed at such frequencies.

### 2.4 Electromechanical Conversion Performance of PCPNs

**Figure 5:**
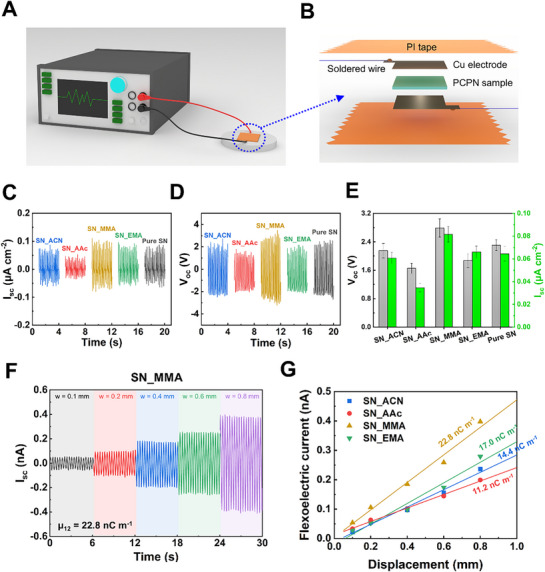
a) Schematic illustration of the electromechanical conversion testing setup and b) the fabricated PCPN devices. c) Open‐circuit voltage and d) short‐circuit current generated by PCPNs under finger tapping. e) Summary of the electromechanical conversion performance of the PCPN samples under finger tapping. f) Flexoelectric current of SN_MMA under cyclic bending with varying mechanical deflection. g) Linearly fitted flexoelectric current as a function of mechanical displacement for PCPN samples.

The responses of the PCPN samples were experimentally measured to evaluate how these structural and molecular factors influence actual electromechanical behavior. As illustrated in **Figure** **5**a, a mechanical force was applied to each sample while the resulting electrical signals were recorded using a multimeter. For consistent measurement conditions, each PCPN film was placed between two copper electrodes connected with soldered wires and encapsulated with polyimide tape, providing electrical stability (Figure 5b).^[^ ^42^ ^]^ The open‐circuit voltage and short‐circuit current generated under repeated finger tapping are presented in Figure 5c–e. Finger tapping of the SN_MMA device produced the highest open‐circuit voltage of 2.79 V and a short‐circuit current density of 0.082 µA cm^−2^ among all PCPNs and pure SN. This superior performance can be attributed to the synergistic effect of favorable morphology and the rotational freedom of SN molecules. In contrast, SN_AAc exhibited the lowest output values (1.65 V and 0.035 µA cm^−2^), indicating that although having a favorable morphology for efficient stress localization, the restricted reorientational motion of SN at the polymer–SN interface severely suppressed polarization in the PCPN system. Given that SN_ACN (2.15 V, 0.061 µA cm^−2^) showed a higher performance compared to SN_AAc, this observation suggests that the reorientational motion of SN is a more critical factor for electromechanical performance than its conformational inversion. Finally, SN_EMA (1.87 V, 0.066 µA cm^−2^) exhibited slightly lower performance than pure SN (2.30 V, 0.064 µA cm^−2^), likely due to the lower SN content and the limited benefit of morphological effects. Hand tapping produced similar trends across all samples, with SN_MMA showing the highest output, reaching up to 15.2 V and 0.48 µA cm^−2^ (Figure S11, Supporting Information).

To verify the presence of reliable flexoelectric behavior, the electrical responses of the PCPNs were evaluated as a function of applied strain gradient (Figure 5f and Figure S12, Supporting Information). The flexoelectric coefficient (*µ_12_*) of the PCPNs upon bending was derived from Equation 3:

(3) $$i=\frac{12\pi fb\mu_{12}}{L}w$$

where *w* is the displacement at the film center, *b* is the width, *L* is the length, *f* is the film bending frequency (fixed at ≈3 Hz), and *i* is the flexoelectric current generated during film bending cycles.^[^ ^43^, ^44^ ^]^ Figure 5g shows the linear relationship between film‐center displacement and the generated flexoelectric current. The linear fit yielded a high coefficient of determination (R^2^ > 0.96), confirming the reliability of the measured flexoelectric response. Based on the slope of the linear fit, the calculated flexoelectric coefficient was highest for SN_MMA (22.8 nC m^−1^), followed by SN_EMA (17.0 nC m^−1^), SN_ACN (14.4 nC m^−1^), and SN_AAc (11.2 nC m^−1^), consistent with their voltage outputs under mechanical loading. The strong linearity and strain‐gradient dependence observed in the PCPNs confirm that the measured signal originates from genuine flexoelectric coupling, further supported by the high signal reproducibility and the well‐behaved proportional increase in current with increasing mechanical deformation.

In addition, to determine the optimum flexoelectric coefficient, MMA‐based PCPN samples with varying SN contents were fabricated and evaluated (designated as SN_MMA_k wt.%, where k denotes the SN content; SN_MMA_58.3 wt.% is identical to SN_MMA as mentioned earlier). As shown in Figures S13 and S14 (Supporting Information), the flexoelectric coefficient gradually increased from 41.7 to 66.7 wt.%, reaching a maximum of 26.5 nC m^−1^, but reduced significantly to 12.2 nC m^−1^ at 75.0 wt.% SN. This trend is attributed to the sample morphologies, as shown in Figure S15 (Supporting Information), which support our previous hypothesis regarding the correlation between morphology and flexoelectric performance. At higher SN content, specifically 75.0 wt.%, the interconnected polymer network was disrupted, leading to a polymer droplet microstructure that impaired stress transfer and consequently reduced the flexoelectric coefficient. These results confirm that the combination of interfacial morphology and SN mobility in SN_MMA yields the highest intrinsic flexoelectric functionality.

**Figure 6:**
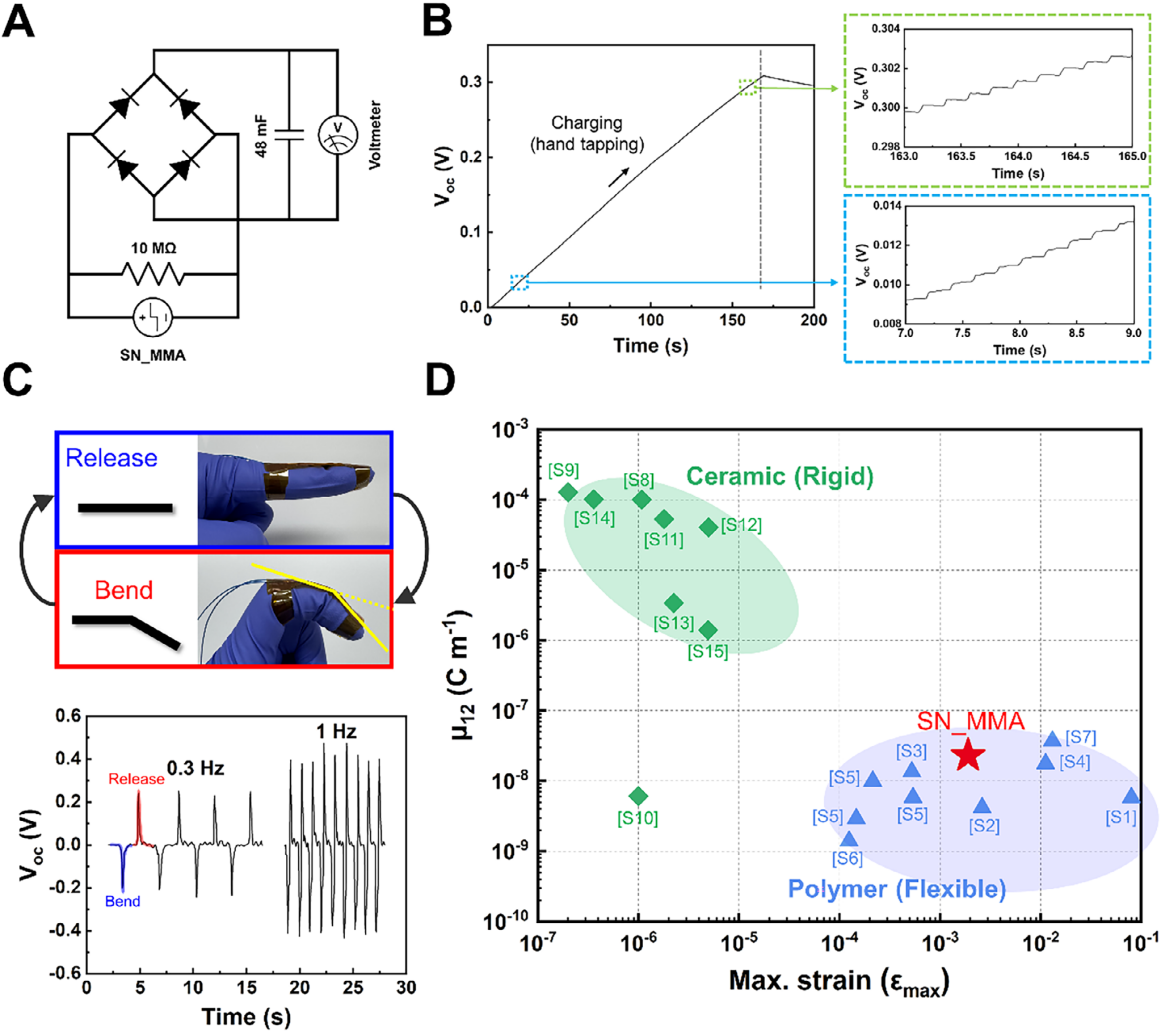
a) Schematic of the rectifier–capacitor charging circuit for the SN_MMA device. b) Charging and discharging curves of the capacitor under external hand tapping applied to SN_MMA. c) Open‐circuit voltage generated by SN_MMA during finger bending and release. d) Comparison of the transverse flexoelectric coefficient (*µ_12_*) and the maximum strain (*ε_max_*) of SN_MMA with those of previously reported materials.

To assess the energy harvesting capability of SN_MMA, the device was connected to a rectifier circuit with a capacitor (**Figure** **6**a). As shown in Figure 6b, electricity generated by hand tapping was successfully accumulated in the capacitor, exhibiting a faster charging rate compared to the discharging rate. Until the voltage reached 0.3 V, the charging curve maintained a stable slope, indicating a consistent and efficient charging behavior. Figure 6c illustrates a sensing test, where a long, rectangular SN_MMA device was attached to a finger and subjected to cyclic bending and releasing motions. Upon bending, the device generates a negative voltage, while releasing induces a positive voltage. Bending the finger to 30° at 0.3 Hz produced 0.22 V, which increased to 0.42 V at 1 Hz. The output signals of SN_MMA were highly stable and reproducible across repeated mechanical cycles, confirming both the mechanical robustness and sensing reliability of the system. To assess long‐term durability, the voltage output was monitored over 10,000 cycles under a constant load of 30 N (Figure S16, Supporting Information). Throughout the test, SN_MMA consistently delivered an output of ≈9.3 V without noticeable performance degradation. The applied load decreased by only 6.14% after 10,000 cycles (Figure S17, Supporting Information), further demonstrating the mechanical integrity of the device. Figure 6d and Table S2 (Supporting Information) present a comparison of SN_MMA with previously reported materials in terms of flexoelectric coefficient and flexibility. Flexibility was evaluated using the maximum strain, calculated from the maximum measured deflection according to the following Equation 4:

(4) $$\varepsilon_{11,max}=k\frac{wd}{L^{2}}$$

where *k* = 24 for cantilever bending, *k* = 6 for three‐point bending, and *k* = 12*A L* ^−1^ for four‐point bending, with A being the distance from the end to the first loading point.^[^ ^44^ ^]^ As shown in Figure 6d, SN_MMA exhibits a relatively high flexoelectric coefficient compared to other polymer‐based materials, positioning it among the upper range within the polymer group. While ceramic materials display flexoelectric coefficients that are 2 to 3 orders of magnitude higher, their intrinsic brittleness and rigidity hinder their use in flexible or deformable systems. Polymers, on the other hand, offer excellent mechanical flexibility and stretchability, which are crucial for wearable or structurally dynamic devices. By combining decent electromechanical responsiveness with the mechanical advantages of polymers, SN_MMA shows potential as a stable and efficient platform for energy harvesting and flexible sensing applications, where long‐term operational stability is essential.

## 3 Conclusion

This study presented a polymer–plastic crystal hybrid strategy for achieving enhanced and reliable flexoelectric electromechanical coupling, using SN as the polar plastic crystalline material. By systematically varying the chemical structure of the polymer network, we revealed that both the composite microstructure and rotational freedom of SN molecules were critical to flexoelectric performance. A heterogeneous polymer morphology enabled localized stress transfer and strain gradient formation, while sufficient molecular mobility allowed SN dipoles to reorient under deformation, thereby promoting polarization. Among the systems investigated, SN_MMA exhibited the most outstanding performance, including the highest electromechanical output and a strong linear relationship between electrical response and applied strain gradient. This result reflected the favorable balance in SN_MMA between interfacial structure and molecular dynamics. The polymer domains in this system are finely dispersed yet not overly restrictive, enabling both mechanical stress localization and dipole mobility. The device also demonstrated excellent durability, maintaining a consistent voltage output over 10,000 loading cycles without noticeable degradation. Nevertheless, its thermal stability remains limited, as the plastic crystalline phase of SN exists within a temperature range of −35 to 62 °C. This limitation can be mitigated by selecting plastic crystals with phase transition temperatures appropriately matched to the operational conditions required for each application. We believe that these findings provide fundamental guidance for designing next‐generation flexoelectric materials. Carefully tuning the interaction between dynamic dipolar molecules and a mechanically structured matrix makes it possible to develop energy harvesting and sensing platforms that combine high performance with long‐term operational stability.

## 4 Experimental Section

### Materials

Succinonitrile (SN; > 99.0%), ethyl methacrylate (EMA; > 99.0%), and methyl methacrylate (MMA; > 99.8%) were purchased from Tokyo Chemical Industry Co., Ltd. (Tokyo, Japan). Acrylic acid (AAc; ≥ 99.0%), Acrylonitrile (ACN; ≥ 99%), trimethylolpropane ethoxylate triacrylate (TRA; Mn ∼692), and 1‐hydroxycyclohexyl phenyl ketone (PI184; 99%) were obtained from Sigma–Aldrich (St. Louis, MO, USA). All chemicals were used as received without further purification.

### Fabrication of Plastic Crystal–Polymer Networks

PCPNs were fabricated via one‐pot free radical polymerization of each monomer (EMA, MMA, AAc and ACN) and crosslinker (TRA) in the presence of SN. Monomer solutions were prepared by mixing 1.2 g of the monomer, 2.1 g of SN, 0.3 g of TRA, 24 mg of PI184. The mixture was stirred until fully dissolved, then degassed by bubbling with Ar gas for 30 min. The resulting solution was poured into a 4 × 6 cm^2^ PTFE mold and UV cured (365 nm) for 1 h. The mold was covered with a glass plate to prevent monomer evaporation during curing. Consequently, bulk films with 1.0 mm thickness were obtained and were cut into desired dimensions for subsequent measurements. The fabricated samples containing SN were designated as SN_x, where x represents the monomer used. For example, the PCPN sample based on MMA was denoted as SN_MMA.

### Material Characterization

The mechanical properties of the samples were evaluated using a motorized force tester (MultiTest 2.5‐dV(u), Mecmesin) equipped with an ELS 500 N load sensor. For compression tests, cylindrical samples (diameter = 2.5 cm, height = 0.7 cm) were compressed at a crosshead speed of 2 mm min^−1^. Loading–unloading cyclic tests were performed at the same crosshead speed with a 10% strain for five cycles. The recovery ratio was defined as the ratio of the maximum stress in each cycle to that of the first cycle. FT‐IR spectroscopy in attenuated total reflectance (ATR) mode was conducted over the 4000–400 cm^−1^ spectral range with 64 scans and a resolution of 4 cm^−1^ (Nicolet iS50, Thermo Fisher Scientific). DSC was performed in an N_2_ atmosphere over a temperature range from –80 °C to 80 °C at a 10 °C min^−1^ heating rate (DSC 25, TA Instruments). PXRD patterns were collected using a θ–2θ scan over 5–50° with Cu Kα radiation (λ = 0.154 nm) (D/MAX‐2500 V/PC, Rigaku). Cross‐sectional morphologies of the samples were observed using an SEM (CX‐200, COXEM). The samples were kept under vacuum for 5 days before SEM measurement to sublimate residual SN and ensure a stable high‐vacuum environment during analysis. The complex permittivity of the samples was measured using a broadband dielectric spectrometer (CONCEPT 40, Novocontrol GmbH) over a 10^6^–10^−1^ Hz frequency range. Dispersion‐corrected density functional theory (DFT‐D) calculations were performed using the DMol3 module in the Materials Studio package (BIOVIA). The generalized gradient approximation (GGA) with the Perdew–Burke–Ernzerhof (PBE) functional and the DNP 3.5 basis set was employed. Grimme's dispersion correction was applied.

### Evaluation of Electrochemical Coupling

First, the PCPN samples were cut into square shapes with dimensions of 10 × 10 mm^2^. Copper plates were attached to both sides of the sample to form a sandwich structure and compressed under 1 MPa to eliminate interfacial air gaps. Electrical wires were then soldered to each copper plate, and the assembly was encapsulated with polyimide tape to prevent delamination and ensure stable contact. For comparison, a pure SN sample was prepared by compressing SN into transparent films, using the same electrode configuration as that used for the PCPNs. Subsequently, the open‐circuit voltage and short‐circuit current density were measured under mechanical stimulation (finger tapping and hand tapping) using a digital multimeter (DMM7510, Keithley, Tektronix).

For the evaluation of the flexoelectric coefficient, PCPN samples were cut into rectangular shapes with dimensions of 55 × 10 mm^2^. The remaining fabrication steps were identical to those described above. The fabricated devices were mounted in a dynamic mechanical analyzer (DMA 850, TA Instruments) and subjected to cyclic three‐point bending at a fixed frequency of 3 Hz. The electrical response was recorded using the same multimeter.

For cyclic durability testing, a programmable mechanical cycling system (SnM‐pp (Push & Pull) machine, SnM) was used to apply 10,000 cycles of constant pressure.

## Conflict of Interest

The authors declare no conflict of interest.

## Supporting information



Supporting Information

## Data Availability

The data that support the findings of this study are available from the corresponding author upon reasonable request.
