# Supplementary material for: Flexoelectric Elastomer Enabled by Miscibility‐Driven Succinonitrile Molecular Rotation
Source: Adv Sci (Weinh). 2025 Nov 7;13(5):e20317. doi: 10.1002/advs.202520317 (PMC12849880; doi:10.1002/advs.202520317)
Supplement: Supplementary file 1 — Supporting Information [file ADVS-13-e20317-s001.docx]

Supporting Information

**Flexoelectric Elastomer Enabled by Miscibility-Driven Succinonitrile Molecular Rotation**

Moonseok Jang^†^, Bitgaram Kim^†^, and Ji-Hun Seo^*^

Department of Materials Science and Engineering, Korea University, 145 Anam-ro, Seongbuk-gu, Seoul 02841, Republic of Korea

Corresponding Author

* Ji-Hun Seo − Email: seojh79@korea.ac.kr


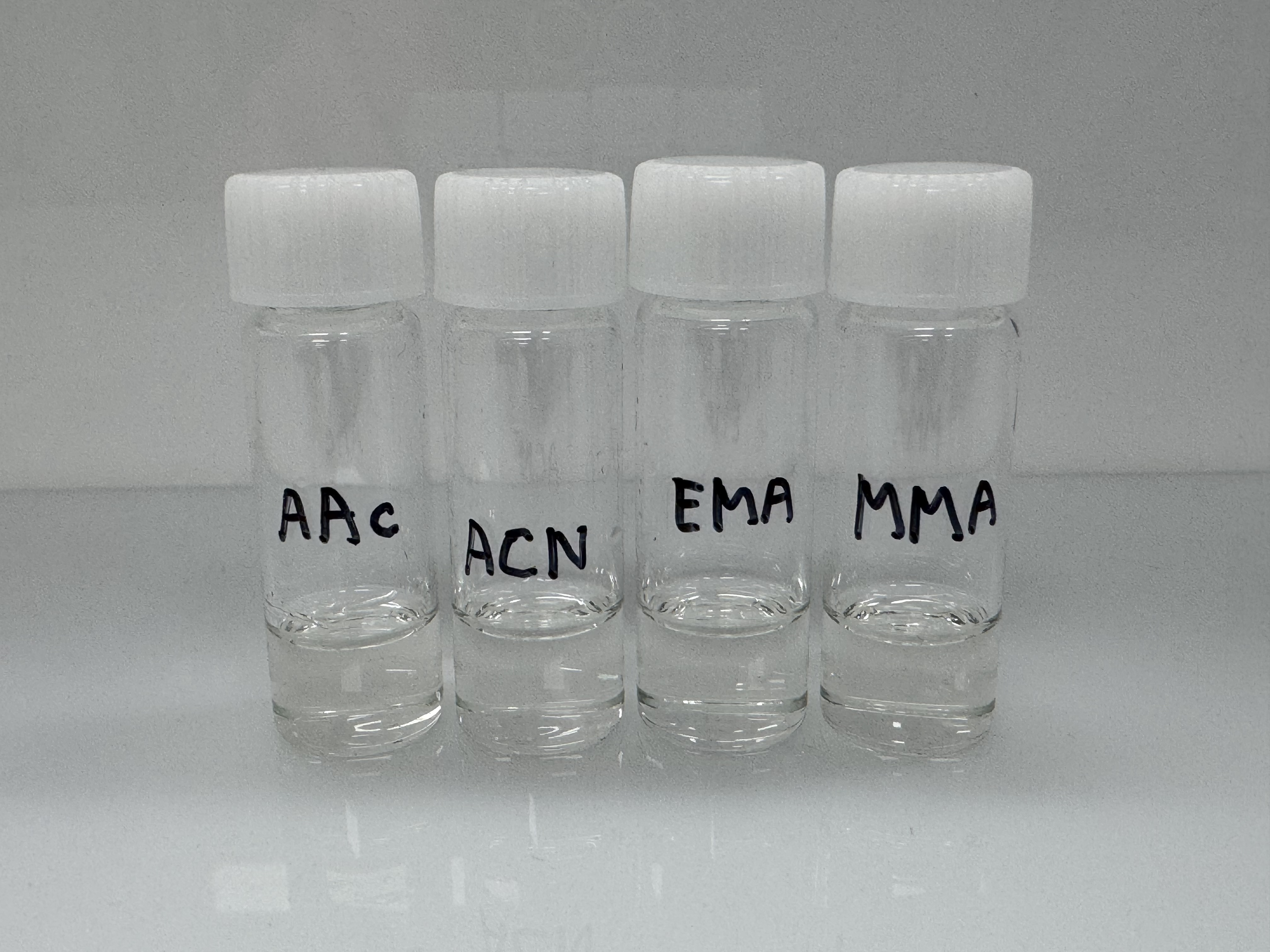


Figure S1. Optical images of monomer–SN solutions. All monomers clearly dissolved 58wt% of SN.

Table S1. Solubility of succinonitrile in the monomers used in this study.

| **Monomers** | **Solubility of succinonitrile** |
| --- | --- |
| Acrylonitrile (ACN) | > 4.2 g mL^-1^ |
| Acrylic acid (AAc) | > 2.5 g mL^-1^ |
| Methyl methacrylate (MMA) | > 2.5 g mL^-1^ |
| Ethyl methacrylate (EMA) | 1.91 g mL^-1^ |


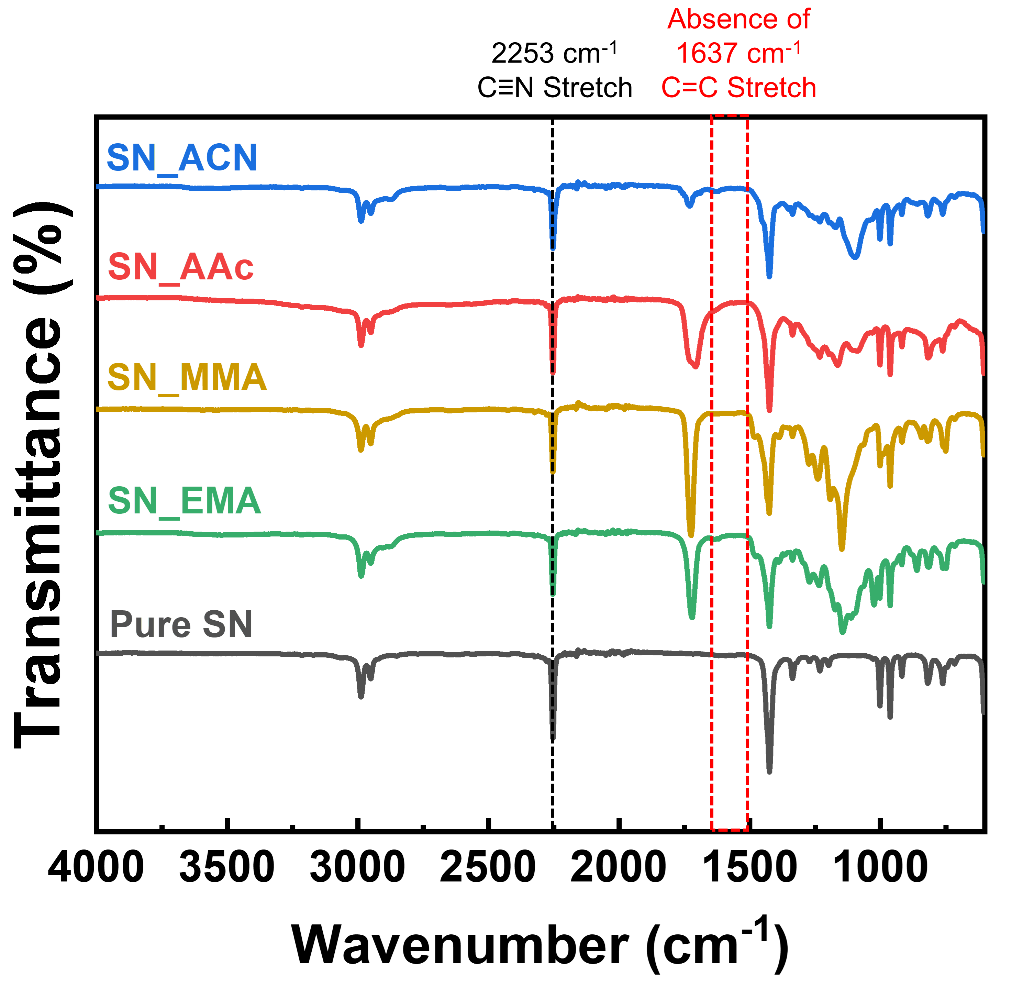


Figure S2. ATR FT-IR spectra of fabricated PCPN films and pure SN.


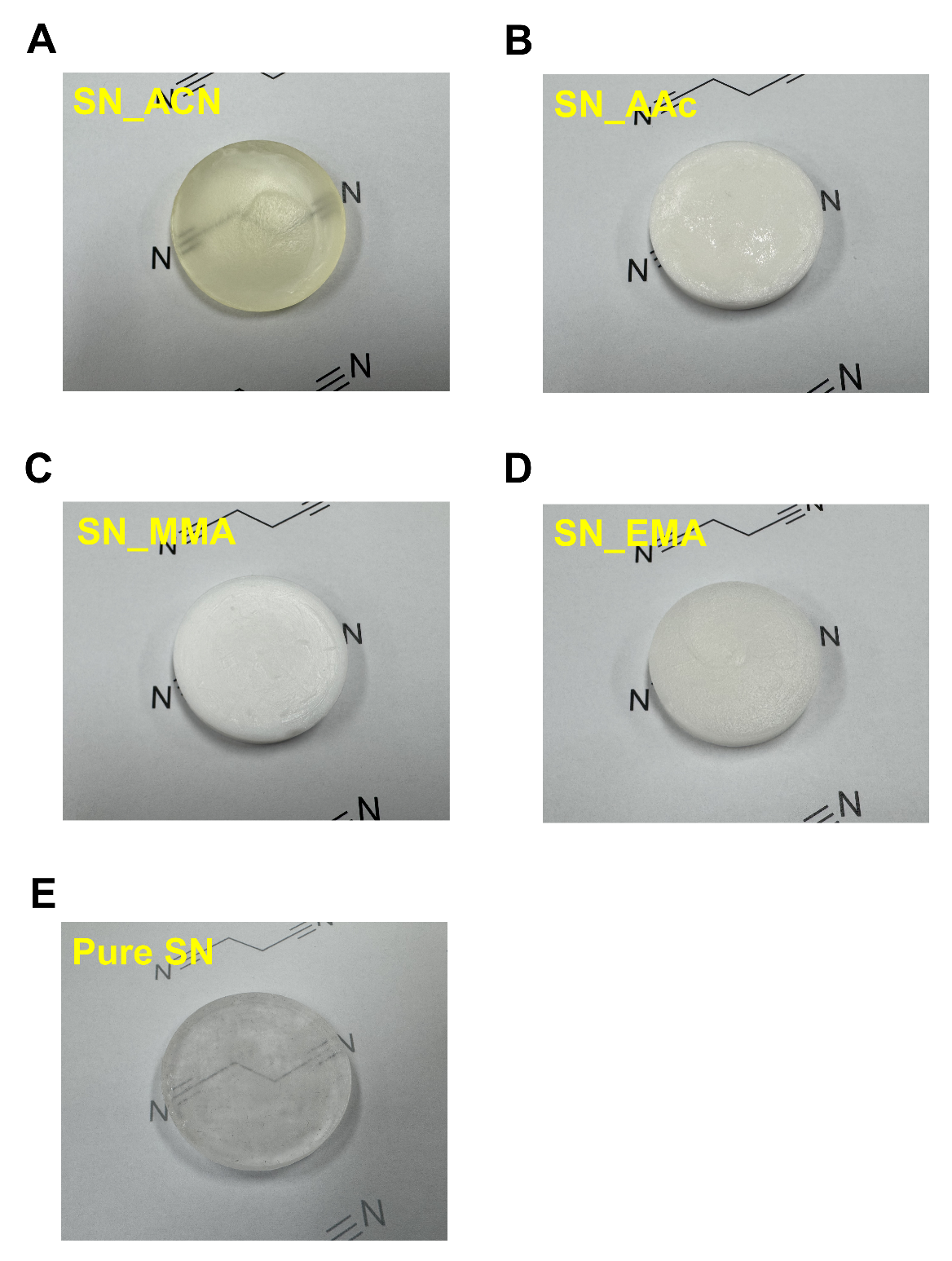


Figure S3. Optical images of fabricated PCPNs: a) SN_ACN, b) SN_AAc, c) SN_MMA, d) SN_EMA, and e) pure SN.


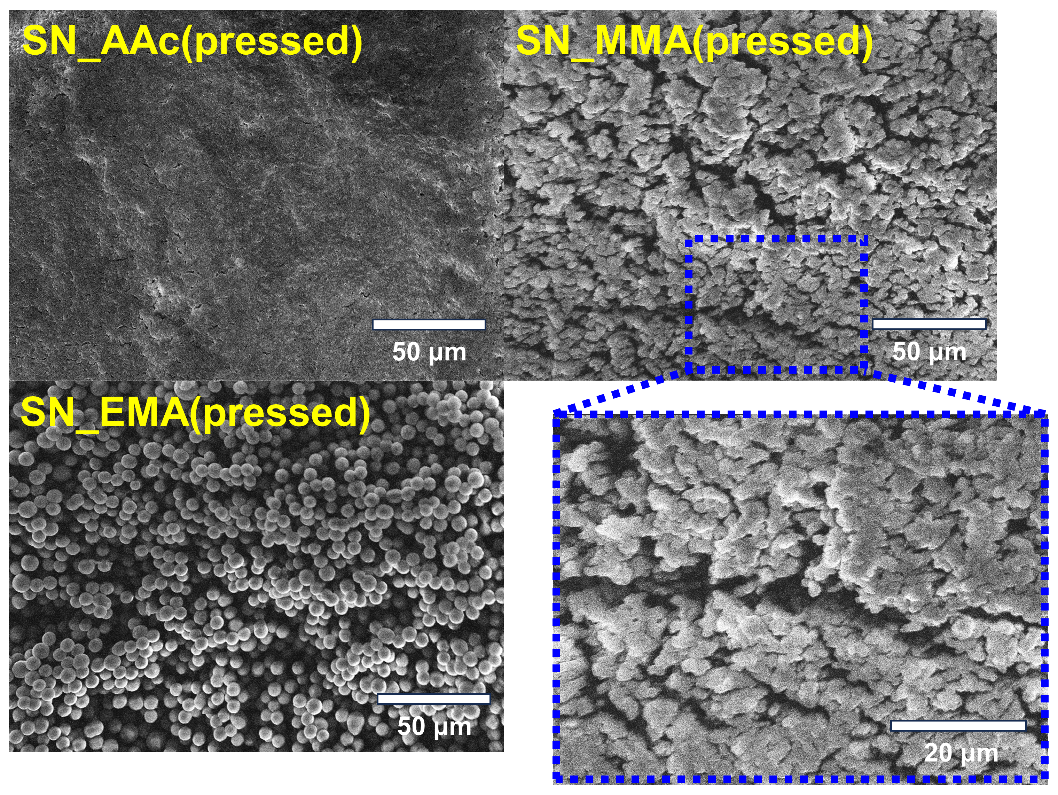


Figure S4. Cross-sectional SEM images of pressed(1 GPa) PCPN samples.


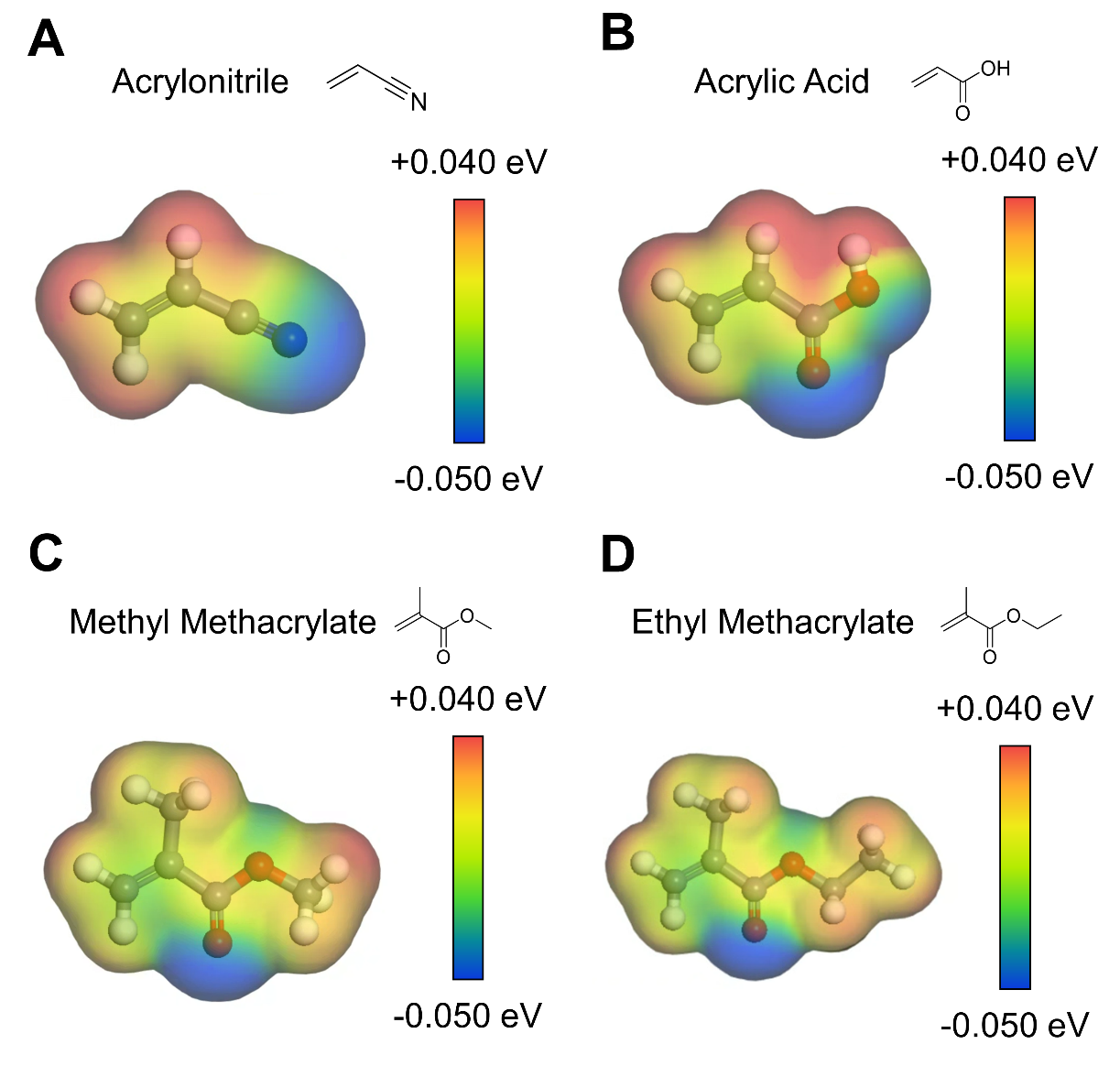


Figure S5. Electrostatic potential calculation of used monomers: a) acrylonitrile (ACN), b) acrylic acid (AAc), c) methyl methacrylate (MMA), and d) ethyl methacrylate (EMA).


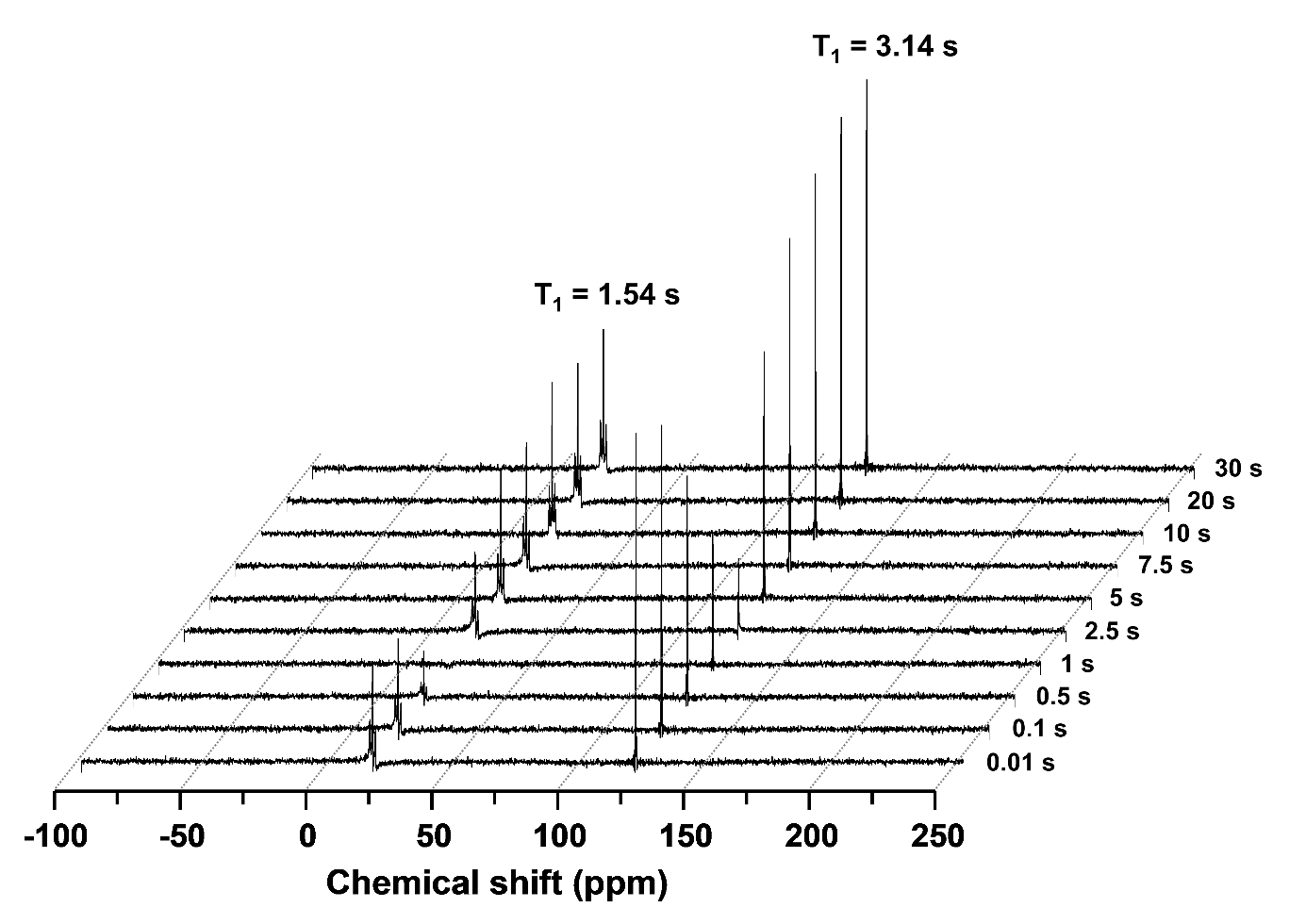


**Figure S6.** ^13^C NMR spin-lattice relaxation of pure SN obtained by the inversion-recovery pulse sequence method.


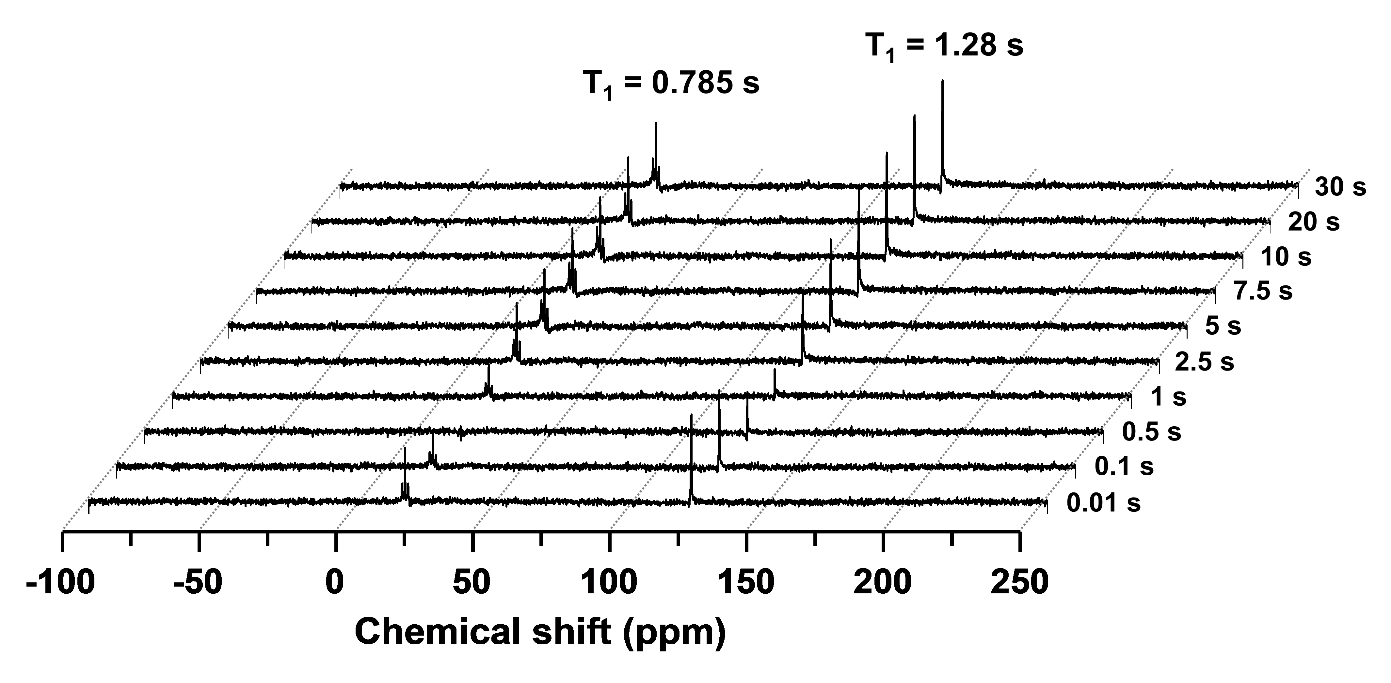

**Figure S7.** ^13^C NMR spin-lattice relaxation of SN_ACN obtained by the inversion-recovery pulse sequence method.


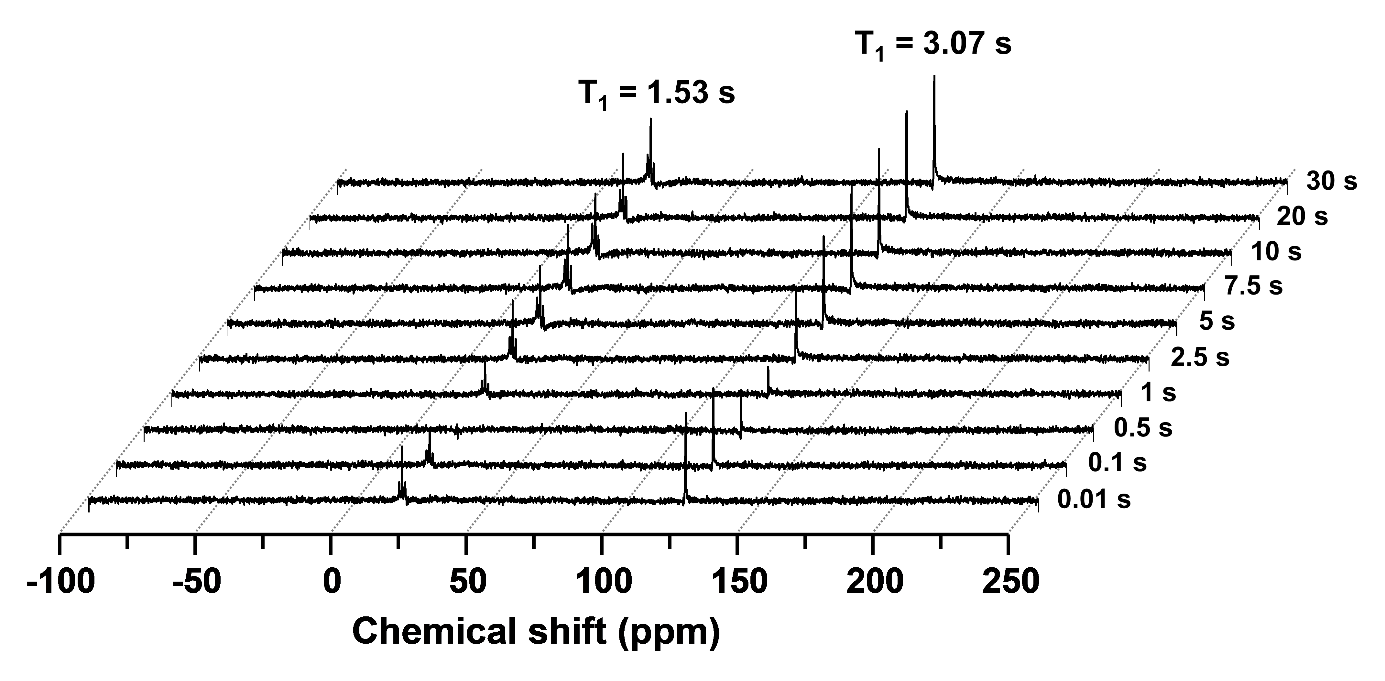


**Figure S8.** ^13^C NMR spin-lattice relaxation of SN_AAc obtained by the inversion-recovery pulse sequence method.


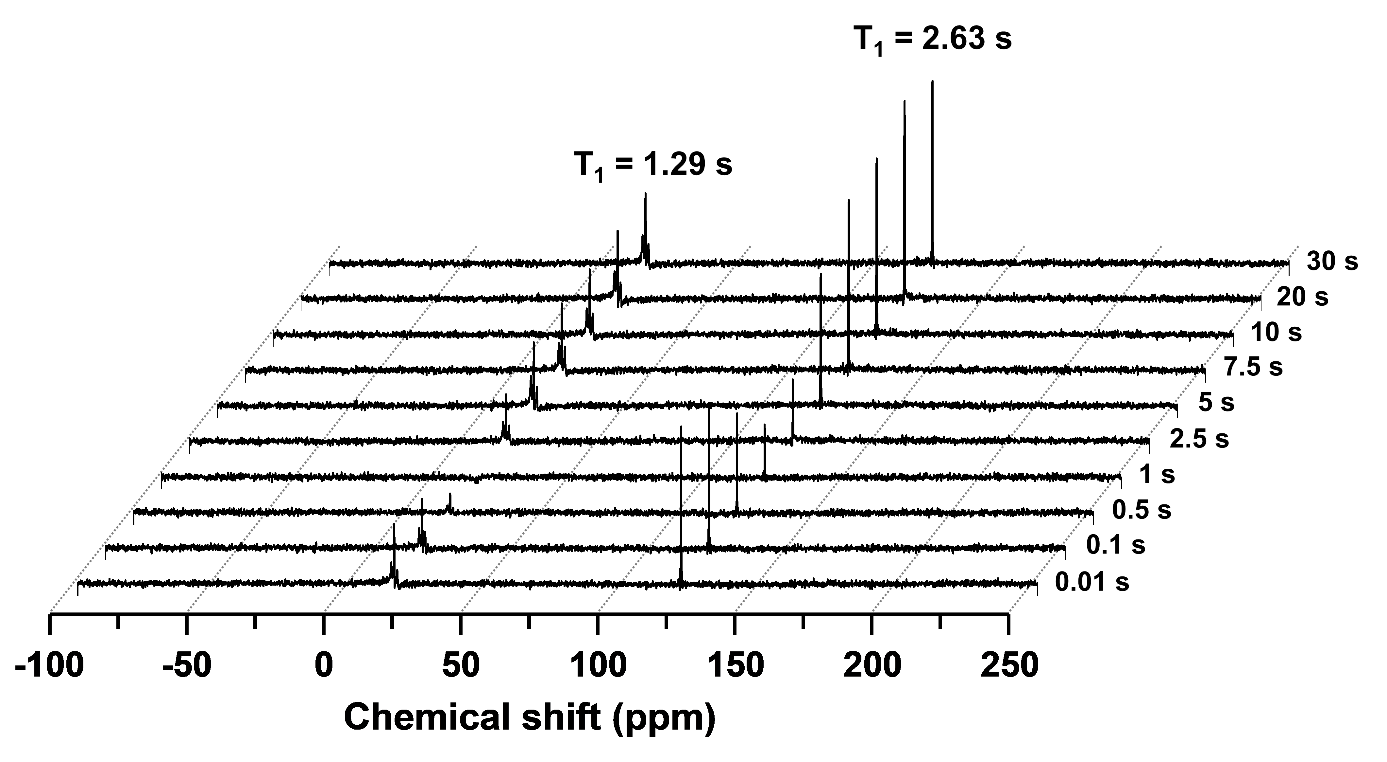


**Figure S9.** ^13^C NMR spin-lattice relaxation of SN_MMA obtained by the inversion-recovery pulse sequence method.


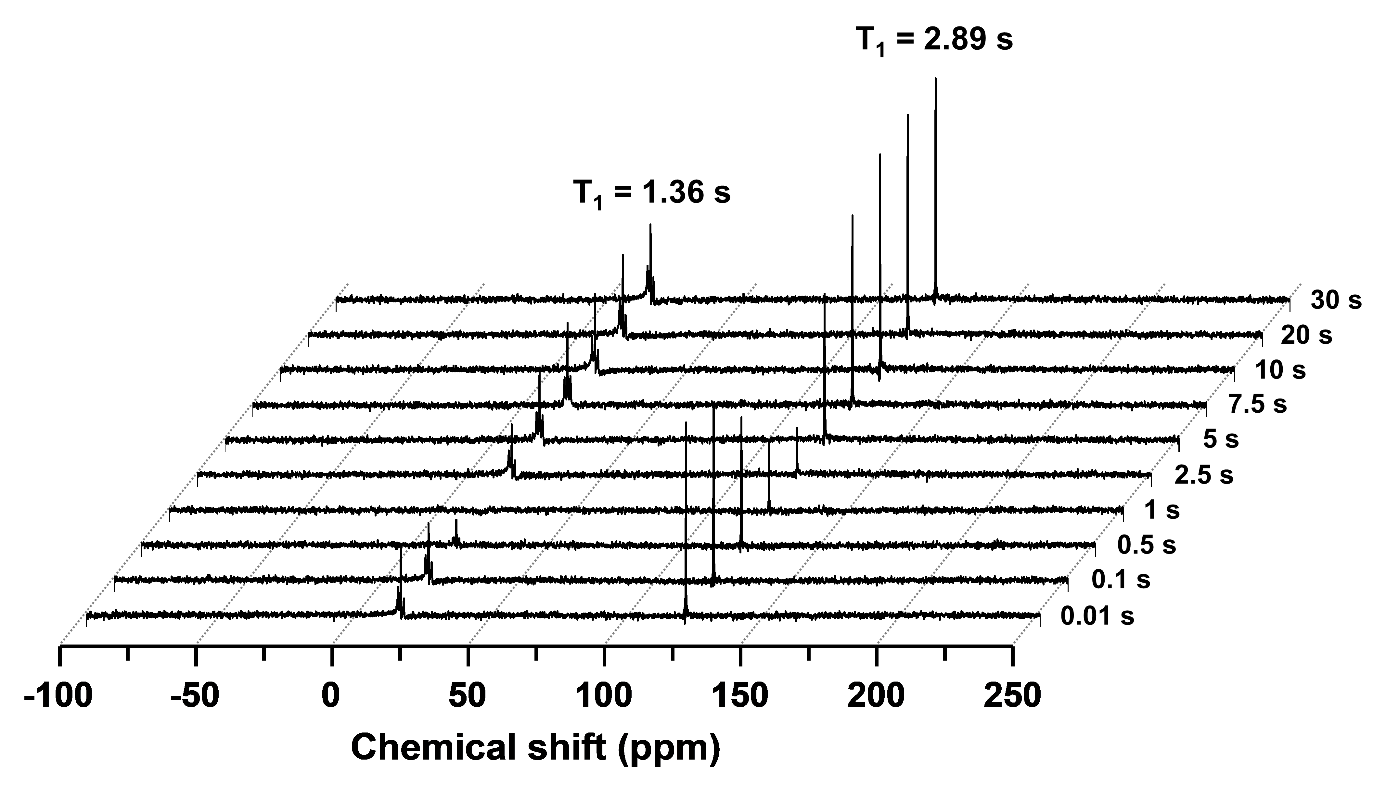


**Figure S10.** ^13^C NMR spin-lattice relaxation of SN_EMA obtained by the inversion-recovery pulse sequence method.
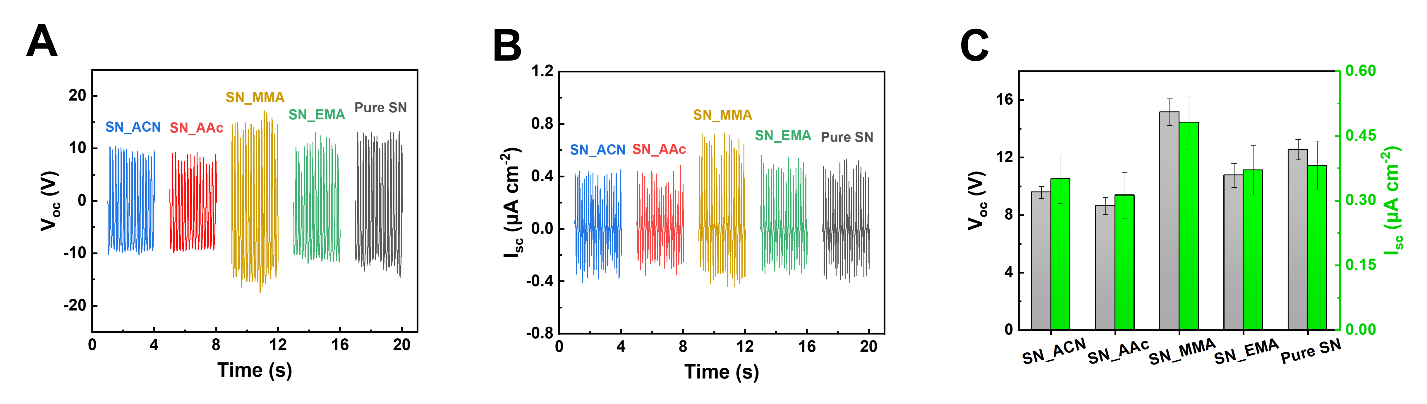


Figure S11. a) Open circuit voltage and b) short circuit current generated by PCPNs under hand tapping. c) Summary of the electromechanical conversion performance of the PCPN samples under hand tapping.


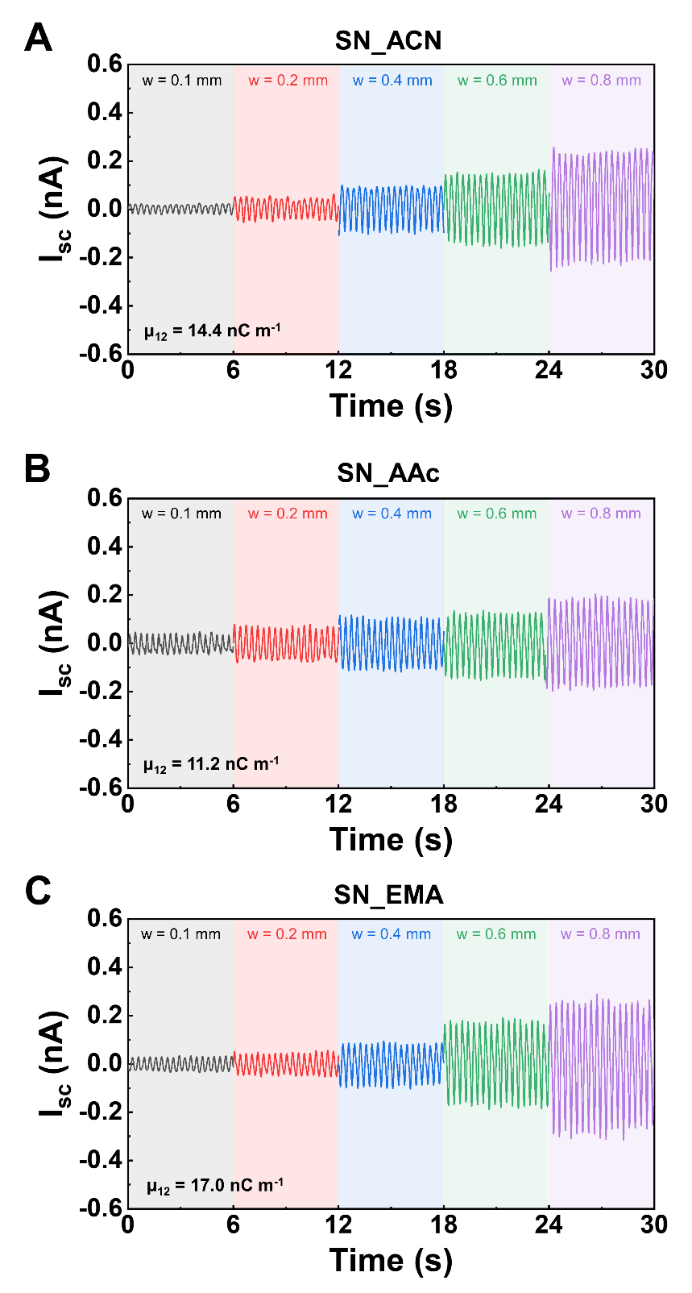


**Figure S12.** Flexoelectric current of a) SN_ACN, b) SN_AAc, and c) SN_EMA under cyclic bending with varying mechanical deflection.


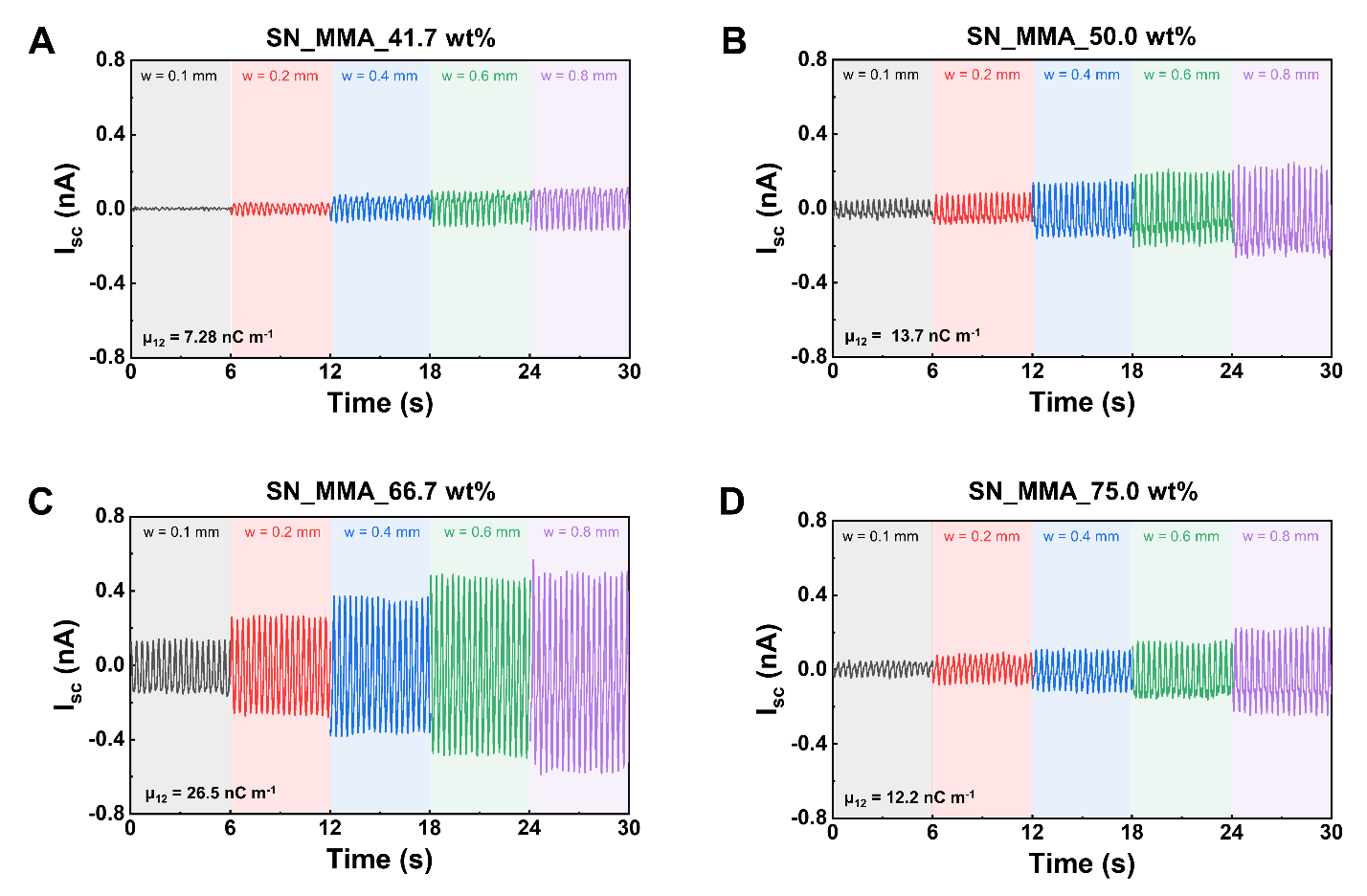


**Figure S13.** Flexoelectric current of a) SN_MMA_41.7 wt%, b) SN_MMA_50 wt%, c) SN_MMA_66.7 wt%, and d) SN_MMA_75 wt% under cyclic bending with varying mechanical deflection.


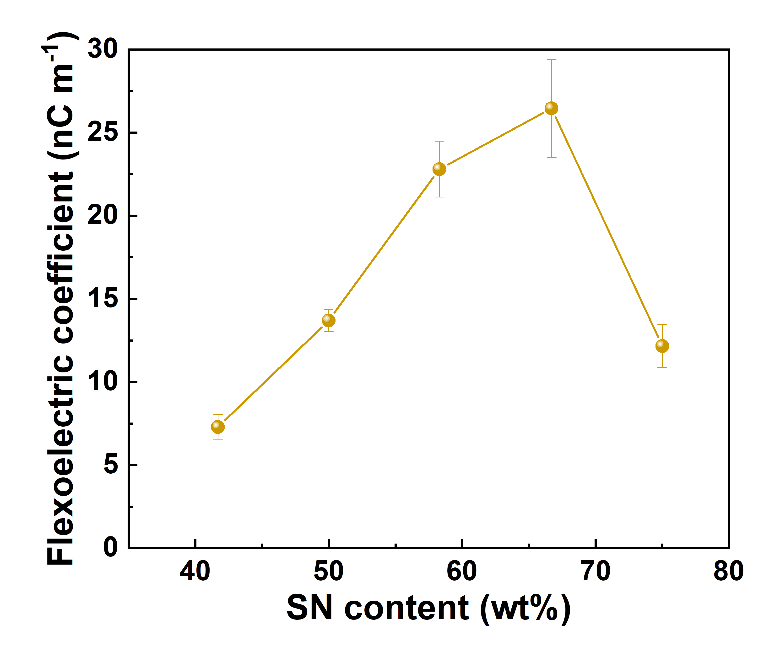


**Figure S14.** Flexoelectric coefficients of MMA-based PCPNs with varying SN contents.


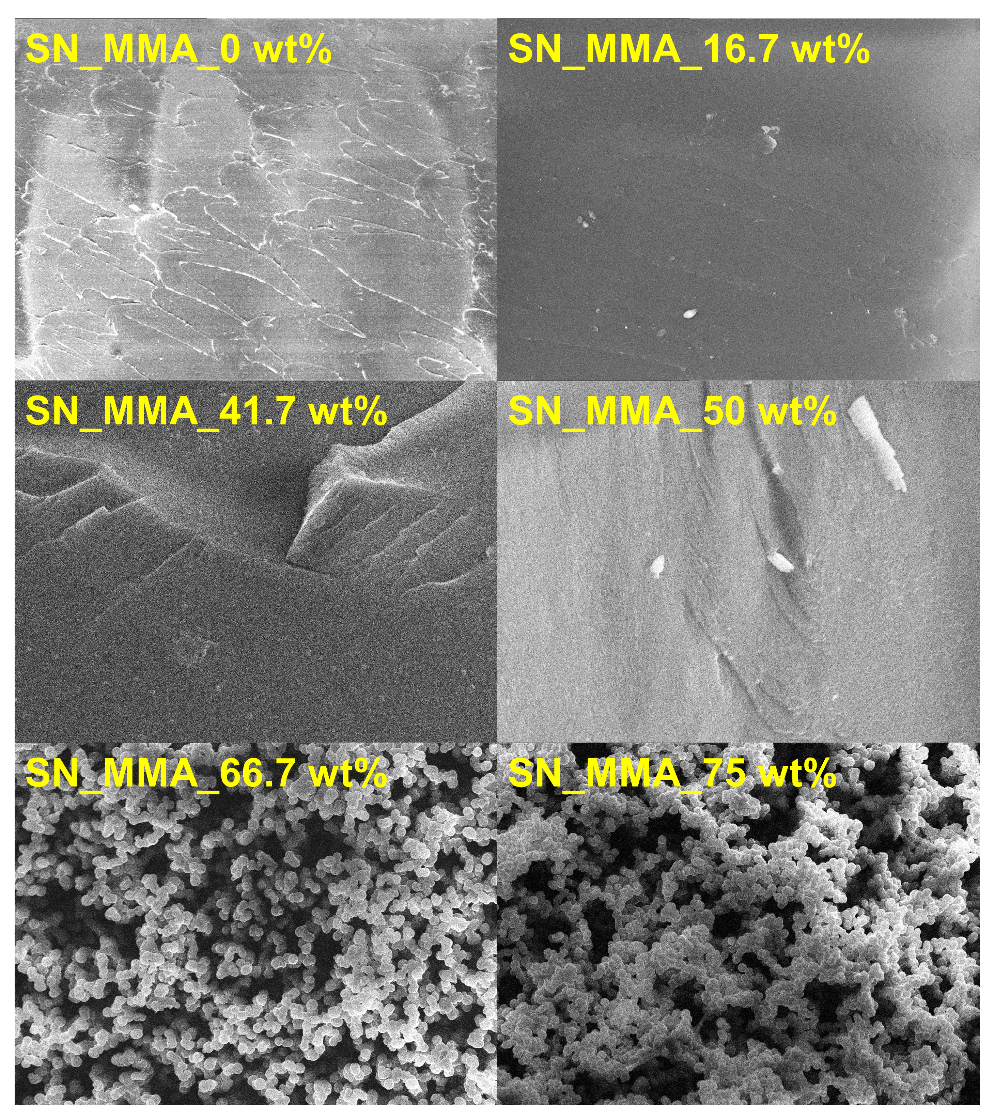


**Figure S15.** Cross-sectional SEM images of MMA-based PCPNs with varying SN contents.


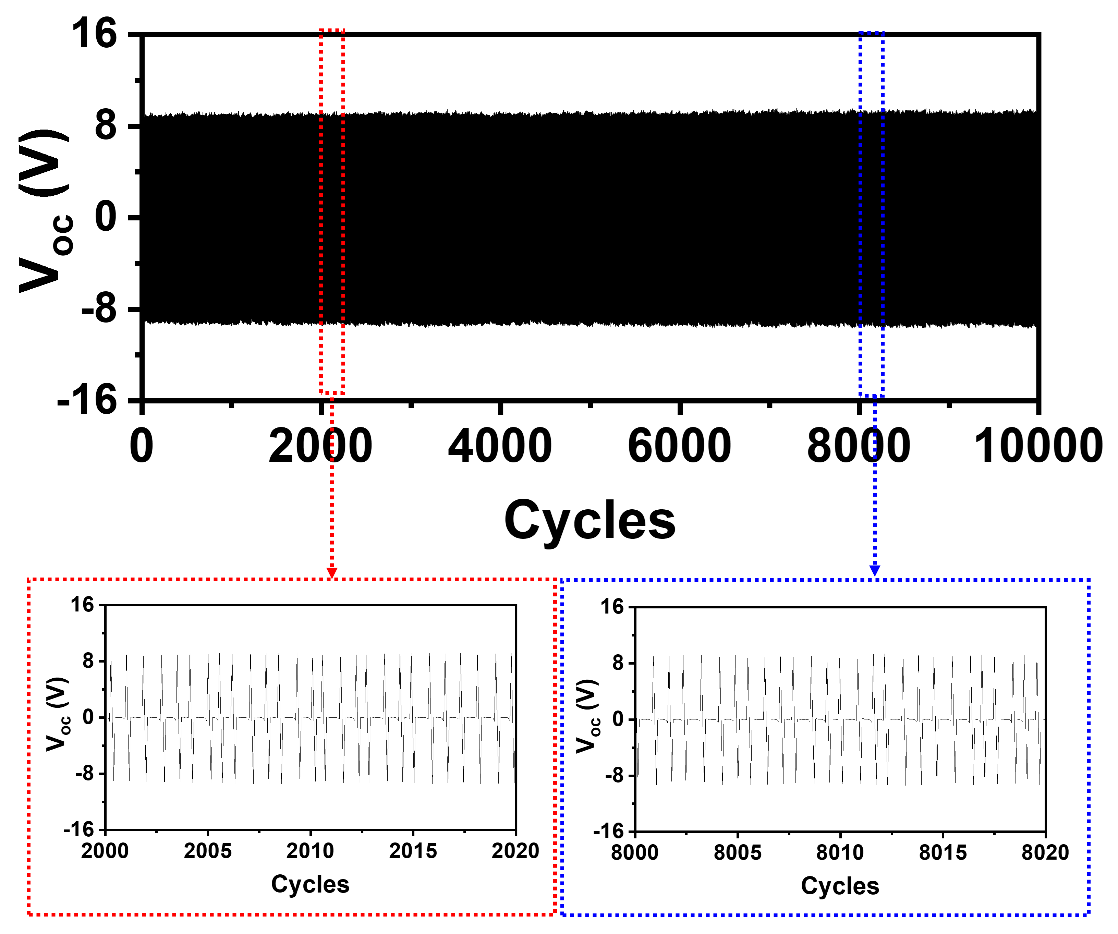


Figure S16. Cyclic durability of SN_MMA under 10,000 loading cycles at 50 N.


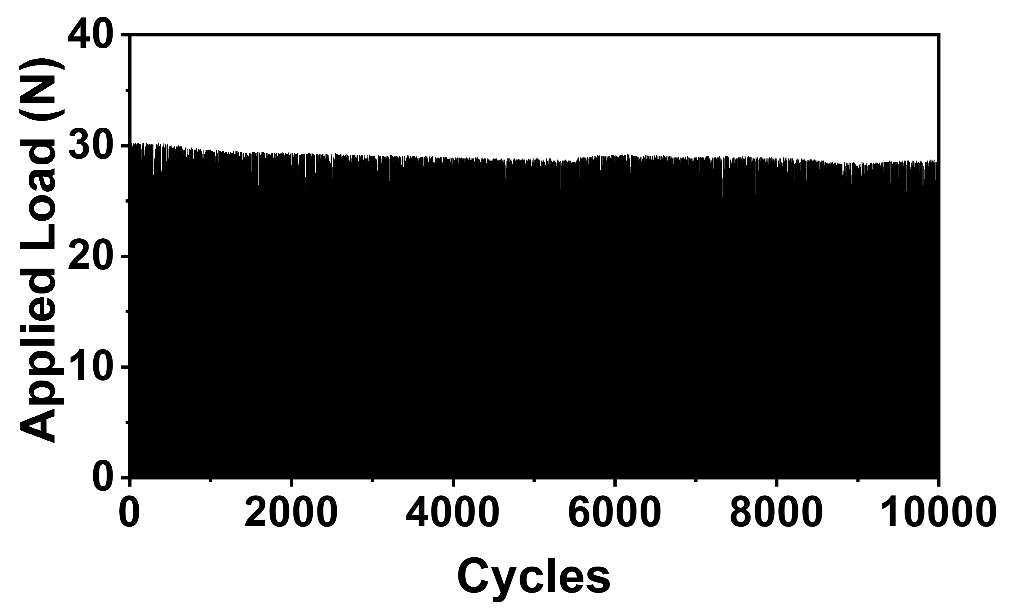


Figure S17. Applied cyclic loading (30 N, 10,000 cycles) during the durability test of SN_MMA.

**Table S2**. Summary of transverse flexoelectric coefficients (*μ*_12_) and strain (*ε*_11_) reported in the current literature.

| **Flexible materials (ε_11_ > 10^-4^)** | | | |
| --- | --- | --- | --- |
| **Material** | **Flexoelectric Coefficient**  **(μC m^-1^)** | **Method** | **Reference** |
| SN_MMA | 0.0228 | 3PB^a^ | This work |
| PVDF | 0.0058 | CB^b^ | [S1] |
| P(VDF-CTFE) | 0.00216 | CB | [S1] |
| P(VDF-HFP) | 0.00257 | CB | [S1] |
| P(VDF-TrFE) (70/30) | 0.00304 | 3PB | [S2] |
| P(VDF-TrFE) (55/45) | 0.00418 | 3PB | [S2] |
| P(VDF-TrFE-CTFE) | 0.00352 | 3PB | [S2] |
| PVDF | 0.00317 | 3PB | [S3] |
| PVDF + 25vol% BST | 0.0135 | 3PB | [S3] |
| PEEK | 0.0176 | CB | [S4] |
| PVDF | 0.013 | CB | [S5] |
| Oriented PET | 0.0099 | CB | [S5] |
| PE | 0.0058 | CB | [S5] |
| Epoxy | 0.0029 | CB | [S5] |
| PDMS | 0.0014 | 3PB | [S6] |
| Porous PVDF | 0.037 | 3PB | [S7] |
| **Rigid materials (ε_11_ < 10^-5^)** | | | |
| **Material** | **Flexoelectric coefficient**  **(μC m^-1^)** | **Method** | **Reference** |
| Ba_0.67_Sr_0.33_TiO_3_ | 100 | CB | [S8] |
| Ba_0.6_Sr_0.4_TiO_3_/Ni_0.8_Zn_0.2_Fe_2_O_4_ | 128 | CB | [S9] |
| SrTiO_3_ | 0.0061 | 3PB | [S10] |
| Ba(Ti_0.87_Sn_0.13_)O_3_ | 53 | CB | [S11] |
| Ba(Ti_0.85_Sn_0.15_)O_3_ | 18.5 | CB | [S12] |
| 0.5wt% Al_2_O_3_-doped Ba(Ti_0.85_Sn_0.15_)O_3_ | 40.5 | CB | [S12] |
| 0.2wt% Al_2_O_3_-doped Ba(Ti_0.85_Sn_0.15_)O_3_ | 2 | CB | [S12] |
| PMN | 3.4 | CB | [S13] |
| PMN-PT | 101 | CB | [S14] |
| PZT | 1.4 | CB | [S15] |

a) Three-point bending

b) Cantilever beam bending

**References**

[S1] Y. Zhou, J. Liu, X. Hu, B. Chu, S. Chen, D. Salem, *IEEE Trans. Dielectr. Electr. Insul.* **2017**, 24, 727.

[S2] J. Liu, Y. Zhou, X. Hu, B. Chu, *Appl. Phys. Lett.* **2018**, 112.

[S3] X. Hu, Y. Zhou, J. Liu, B. Chu, *J. Appl. Phys.* **2018**, 123.

[S4] C. L. Zhang, J. J. Wu, C. W. Wu, Z. Z. He, D. J. Zhu, W. Q. Chen, *Appl. Phys. Lett.* **2024**, 125.

[S5] B. Chu, D. R. Salem, *Appl. Phys. Lett.* **2012**, 101.

[S6] H. Ji, S. Shao, K. Liu, H. Shang, Y. Zhu, T. Wu, S. Shen, S. Zhang, M. Xu, *Appl. Phys. Lett.* **2021**, 119.

[S7] M. Zhang, D. Yan, J. Wang, L.-H. Shao, *J. Mech. Phys. Solids* **2021**, 151, 104396.

[S8] W. Ma, L. E. Cross, *Appl. Phys. Lett.* **2002**, 81, 3440.

[S9] Y. Li, L. Shu, W. Huang, X. Jiang, H. Wang, *Appl. Phys. Lett.* **2014**, 105.

[S10] P. Zubko, G. Catalan, A. Buckley, P. R. L. Welche, J. F. Scott, *Phys. Rev. Lett.* **2007**, 99, 167601.

[S11] L. Shu, X. Wei, L. Jin, Y. Li, H. Wang, X. Yao, *Appl. Phys. Lett.* **2013**, 102.

[S12] L. Shu, M. Wan, Z. Wang, L. Wang, S. Lei, T. Wang, W. Huang, N. Zhou, Y. Wang, *Appl. Phys. Lett.* **2017**, 110.

[S13] W. Ma, L. E. Cross, *Appl. Phys. Lett.* **2001**, 78, 2920.

[S14] L. Shu, M. Wan, X. Jiang, F. Li, N. Zhou, W. Huang, T. Wang, *AIP Adv.* **2017**, 7.

[S15] W. Ma, L. E. Cross, *Appl. Phys. Lett.* **2005**, *86*.
